# Hybrid Validation of a Quality-Controlled, Waveform-Centered AI Framework with Optional Multi-Sensor Support for Seismic Monitoring

**DOI:** 10.3390/s26103269

**Published:** 2026-05-21

**Authors:** Askar Abdykadyrov, Yerik Alipuly, Maxat Mamadiyarov, Bekbolat Tashev, Akerke Yerkinova, Kalmukhamed Tazhen

**Affiliations:** 1 Department of Electronics, Telecommunications and Space Technologies, Satbayev University, Almaty 050013, Kazakhstan; a.abdykadyrov@satbayev.university (A.A.); k.tazhen@satbayev.university (K.T.); 2 National Scientific Center for Seismological Observations and Research, Ministry of Emergency Situations of the Republic of Kazakhstan, Almaty 050060, Kazakhstan; bekbolat.tashev@seismology.kz (B.T.); yerkinovaa@mail.ru (A.Y.)

**Keywords:** seismic monitoring, earthquake detection, phase picking, graph-aware refinement, quality control, waveform-centered AI, reduced-input robustness, optional multi-sensor support, hazard-related characterization

## Abstract

**Highlights:**

**What are the main findings?**

- A quality-controlled, waveform-centered AI framework improved the balance between event detection, false-alarm control, latency, and phase-picking accuracy under the evaluated Almaty-region benchmark.
- Graph-aware refinement and post-inference quality control contributed complementary benefits, while optional auxiliary sensing was most useful under the most adverse controlled stress-test condition.

**What are the implications of the main findings?**

- Robust seismic monitoring should combine waveform inference with explicit reliability control rather than relying only on raw neural predictions.
- Optional DAS, MEMS, and HR-GNSS branches can be treated as auxiliary architectural extensions, while waveform-only fallback remains essential when synchronized auxiliary data are unavailable.

**Abstract:**

Rapid and reliable seismic monitoring requires accurate waveform inference, together with robustness to noise, incomplete sensing, and unstable predictions. This study investigates a quality-controlled, waveform-centered, AI-assisted framework for seismic event detection, P- and S-phase picking, graph-aware inter-station refinement, and rapid hazard-related characterization. The framework includes optional DAS, MEMS, and high-rate GNSS branches; however, the primary empirical validation is based on real waveform-centered IRIS records from the Almaty seismic region, not on a fully synchronized multimodal field deployment. The dataset includes seven seismic stations, HHZ waveforms sampled at 100 Hz, 219 seismic events, 1260 event traces, and 240 s P-centered windows from 1 January 2023 to 31 December 2024. Optional auxiliary branches are evaluated through controlled branch-availability, reduced-input, fallback, and stress-test scenarios. Under the standard-condition benchmark, the proposed framework achieved a precision of 0.941, recall of 0.932, F1 score of 0.936, false-alarm rate of 0.051, detection latency of 173 ms, and P- and S-pick mean absolute errors of 31 ms and 54 ms. Under controlled low-SNR testing, it retained an F1 score of 0.846. The findings support waveform-centered, quality-controlled monitoring, while broader cross-domain and fully synchronized multimodal validation remain necessary.

## 1. Introduction

Rapid and reliable seismic monitoring is a fundamental requirement for earthquake surveillance, microseismic observation, emergency response, and operational hazard assessment. With the continuing growth of continuous-waveform archives and dense instrumental coverage, seismic monitoring systems are increasingly expected to detect weak events, identify seismic phase arrivals, and provide timely information for rapid situational awareness. Recent reviews show that machine learning and deep learning are now becoming central tools in earthquake seismology because they can improve large-scale seismic data processing, but they also introduce new challenges related to robustness, interpretability, and transferability across sensing conditions [1,2].

Conventional automatic monitoring pipelines are still widely based on trigger-type algorithms and classical phase-picking procedures. Although these methods remain computationally efficient and operationally familiar, their performance often degrades in the presence of low signal-to-noise ratios, emergent onsets, waveform distortion, and nonstationary anthropogenic noise. In practice, these limitations affect not only event declaration but also the quality of subsequent phase association, location, and hazard interpretation. Therefore, improving the reliability of seismic sensing and analysis under noisy and heterogeneous operating conditions remains a relevant scientific and practical task [1,2].

A major methodological shift in recent years has been the use of deep neural networks for waveform-based seismic detection and phase picking. PhaseNet demonstrated that three-component seismic waveforms can be mapped directly to P-arrival, S-arrival, and noise probabilities, enabling accurate automated arrival-time picking without handcrafted feature engineering [3]. Earthquake Transformer further extended this idea by jointly addressing event detection and phase picking within a unified attentive architecture, showing that simultaneous treatment of related tasks can improve performance, especially in noisy microearthquake monitoring scenarios [4]. These studies established a strong foundation for AI-assisted seismic monitoring and confirmed that waveform-centered deep learning can substantially outperform many conventional workflows under realistic monitoring conditions [3,4].

At the same time, the sensing landscape in seismology has become substantially more heterogeneous. In addition to conventional broadband and strong-motion stations, recent studies have explored distributed acoustic sensing (DAS), low-cost MEMS accelerometers, and other complementary modalities for earthquake monitoring. For example, PhaseNet-DAS introduced a semi-supervised strategy for seismic arrival-time picking on DAS recordings and showed that deep learning methods can be adapted to spatial–temporal fiber-optic data despite limited manual labels and strong noise contamination [5]. In parallel, recent low-cost MEMS datasets have demonstrated the growing feasibility of using inexpensive sensor nodes for seismic recording and early warning-oriented studies, thereby expanding the practical scope of distributed seismic sensing [6]. These developments open important opportunities for denser and more resilient monitoring systems, but they also create new problems related to signal representation, synchronization, cross-modal consistency, and inference reliability [5,6].

Another important trend is the transition from isolated single-station inference toward integrated multi-station and multi-task seismic monitoring. Recent graph-based approaches have shown that phase picking, event association, and location can be solved jointly by directly exploiting inter-station relationships and physical consistency between subtasks [7]. However, recent evaluations also indicate that high performance achieved in one domain does not necessarily transfer reliably to another. In particular, machine learning-based phase pickers that perform well on seismic land data may degrade when applied to seismic ocean-bottom recordings unless they are specifically adapted to the target domain [8]. This means that, despite substantial progress, a fully robust framework for AI-assisted multi-sensor seismic monitoring under heterogeneous noise and heterogeneous sensing conditions is still lacking. Therefore, research aimed at developing such a framework for rapid event detection, phase picking, and hazard-related auxiliary characterization is timely and relevant.

The results of this study should be interpreted within a clearly defined validation scope. The primary empirical benchmark is waveform-centered and is based on real IRIS/EarthScope records from the Almaty seismic region. The dataset contains HHZ waveform records sampled at 100 Hz from seven regional seismic stations, 219 seismic events, 1260 event traces, and 240 s analysis windows centered on catalog-reported P-wave arrivals for the period from 1 January 2023 to 31 December 2024. Fully synchronized DAS, MEMS, and high-rate GNSS records were not used as a real multimodal field deployment in the present validation. Instead, these sensing modalities are treated as optional architectural branches and are evaluated through controlled branch-availability, reduced-input fallback, and stress-test scenarios. Therefore, this study presents a hybrid validation of a waveform-centered seismic monitoring framework with optional multi-sensor support rather than a complete empirical validation of simultaneous DAS–MEMS–HR-GNSS–seismic data fusion.

The proposed framework is organized around four analytically separable functions. First, a compact waveform-centered inference backbone performs event detection and P/S phase picking from the mandatory seismic waveform branch. Second, a graph-aware refinement module improves station-level consistency after local waveform inference. Third, a post-inference quality-control layer evaluates posterior confidence, augmentation stability, physical plausibility, and station or branch agreement before outputs are accepted, flagged, or rejected. Fourth, a reduced-input fallback mechanism preserves waveform-only operation when optional auxiliary branches are unavailable, incomplete, unsynchronized, or rejected. Therefore, the comparison with conventional, PhaseNet-like, and Earthquake Transformer-like baselines is interpreted as an operational pipeline comparison, not as evidence that the proposed waveform backbone is inherently superior to all existing neural architectures.

The novelty of this study is therefore defined at the monitoring-framework level rather than at the level of a completely new neural phase picker. PhaseNet and Earthquake Transformer established strong paradigms for waveform-based phase picking and joint detection picking, while graph-aware and quality-control approaches have shown the value of inter-station context and post-inference reliability assessment. The contribution of the present study is to combine these elements into a conservative waveform-centered monitoring framework with validation-only QC thresholds, graph-aware station-consistency refinement, physical-consistency filtering, reduced-input fallback, and optional auxiliary-branch routing. This formulation addresses an operational question: whether a complete monitoring pipeline can provide more stable point-estimate behavior under a defined regional waveform benchmark and controlled degradation protocol.

The main contributions of this work are summarized as follows. First, the study defines a waveform-centered, AI-assisted monitoring framework with optional DAS, MEMS, and high-rate GNSS support while preserving waveform-only execution as the validated empirical core. Second, it specifies a reproducible architecture, including a waveform encoder, event-detection head, P/S phase-picking heads, graph-aware refinement, post-inference QC, and auxiliary rapid-characterization head. Third, it introduces validation-derived QC thresholds for confidence, augmentation stability, physical consistency, and agreement assessment. Fourth, it evaluates the framework using a hybrid protocol based on real Almaty-region IRIS/EarthScope waveform records, controlled C1–C3 perturbation conditions, reduced-input scenarios, optional-branch availability tests, and ablation analysis. Fifth, it interprets the results conservatively as point estimates under a limited event-level test set, without claiming statistically proven superiority or full real-world DAS–MEMS–HR-GNSS–seismic fusion.

The rest of this article is organized as follows. Section 2 reviews recent methods of seismic monitoring based on artificial intelligence and identifies methodological limitations related to waveform inference, multimodal sensing, synchronization, differences in sensor responses, domain shift, and quality control. Section 3 defines the purpose and objectives of the study. Section 4 describes the proposed structure, including waveform representation, model architecture, training configuration, quality-control thresholds, dataset structure, validation protocol, controlled noise conditions, reduced-input operation scenarios, evaluation metrics, and statistical analysis. Section 5 includes comparative testing, reliability assessment, architectural validation, multi-task analysis, and ablation results. Section 6 discusses the implications and limitations of the results, including the limited scope of multimodal validation and the need for more cross-domain tests. Section 7 concludes the article and outlines future work on synchronized real-world multi-sensor validation. To address the reviewer concern regarding scope, the revised study is deliberately focused on the evaluated waveform-centered monitoring core. The manuscript does not attempt to validate all possible aspects of multimodal seismic sensing. Instead, it focuses on three experimentally evaluated components: (i) real waveform-centered detection and P/S phase picking using the Almaty-region HHZ benchmark, (ii) graph-aware refinement and post-inference quality control as reliability mechanisms, and (iii) reduced-input and optional-branch scenarios as controlled architectural tests. This focus prevents the optional DAS, MEMS, and high-rate GNSS branches from being interpreted as a fully validated real-world multimodal deployment.

## 2. Literature Review and Problem Statement

Recent studies confirm that the most active progress in AI-assisted seismology has occurred in earthquake detection, phase picking, event association, and catalog development. Machine learning and deep learning methods have improved event detectability, arrival-time reading, and workflow automation, especially for weak seismic events embedded in noise [9,10]. However, many of these advances have been achieved in task-specific settings, where detection, phase picking, association, location, and rapid characterization are optimized separately. As a result, improvement in one processing stage does not automatically guarantee improved reliability of the complete operational monitoring chain.

A major group of studies has focused on waveform-centered inference. PhaseNet demonstrated that seismic waveforms can be mapped directly to P-arrival, S-arrival, and noise probability sequences, while Earthquake Transformer showed that event detection and phase picking can be treated jointly in an attentive deep learning architecture [3,4]. SCALODEEP further demonstrated that generalized deep learning detection can improve robustness across regional datasets [9]. These methods established strong predictive baselines for waveform-based monitoring. Nevertheless, they mainly address neural inference from seismic waveforms and do not, by themselves, resolve the broader operational requirements of monitoring, such as validation-based threshold selection, reduced-input execution, false-alarm control, unstable-output rejection, and explicit quality-control logic.

Network-aware and multi-station models represent another important direction. EdgePhase and recent graph-based approaches have shown that inter-station information, spatial context, and network geometry can improve phase picking, association, and real-time monitoring [7,11,12]. These studies move AI-based seismic processing closer to the way seismic analysts interpret events across a station network. However, most network-aware methods are still developed for relatively homogeneous seismic networks and do not fully address heterogeneous sensing conditions, missing auxiliary inputs, or branch-level reliability differences between conventional seismic stations, DAS arrays, MEMS accelerometers, and GNSS receivers. Therefore, multi-station processing improves an important part of the problem, but it does not fully solve the problem of robust monitoring under heterogeneous multi-sensor availability.

Recent developments in seismic instrumentation have expanded monitoring beyond conventional broadband and strong-motion stations. Distributed acoustic sensing has enabled dense spatial sampling and AI-based arrival-time picking based on fiber-optic recordings [5]. Low-cost MEMS accelerometers have increased the practical feasibility of distributed and resource-constrained seismic sensing [6]. High-rate GNSS has also been explored as a complementary source for rapid-source or magnitude-related characterization, especially for larger events [13]. DAS-based approaches have further shown potential for magnitude estimation, ground-motion prediction, and integration with early-warning workflows [14,15]. Despite these advances, the physical observables produced by these sensors are not equivalent. Conventional seismometers record ground motion at point stations, DAS measures fiber-coupled strain or the strain rate along a cable, MEMS sensors have different noise floors and installation-dependent responses, and high-rate GNSS provides displacement-related information at lower effective temporal resolutions. These differences make direct multimodal fusion mathematically and physically difficult.

Multi-sensor seismic fusion is not only a data-format problem but also a physical and mathematical representation problem. Conventional seismometers record ground motion-related quantities at point stations, DAS measures fiber-coupled strain or the strain rate over a spatially extended cable, MEMS accelerometers provide acceleration measurements with device- and installation-dependent noise floors, and high-rate GNSS provides displacement-related information at lower effective temporal resolutions. These modalities differ in sampling rate, transfer function, amplitude unit, spatial support, timing precision, installation sensitivity, dynamic range, and noise behavior. Directly forcing these heterogeneous observations into a single raw waveform tensor may therefore create physically misleading representations and obscure modality-specific uncertainty. For this reason, the present framework uses modality-specific preprocessing and permits auxiliary branches to contribute only after synchronization, completeness, amplitude-validity, preprocessing-success, and branch-level reliability checks. This design supports conservative late-stage feature or decision construction, while the empirical validation remains explicitly waveform-centered.

Another unresolved issue is robustness to domain shift and unstable noise. AI-assisted phase pickers that work well in one data region may fail when applied to different acquisition conditions, such as ocean-bottom seismic recordings, regional events with low SNR values, or networks with different sensor characteristics [8]. This limitation is especially important for operational seismic monitoring, where anthropogenic noise, environmental noise, station failures, blurred geometry, and incomplete detection are common. Controlled additive perturbation can be useful for reproducible stress tests, but it cannot fully reproduce the complexity of real seismic noise, which is often colored, nonstationary, and location- and source-dependent. Thus, reliability assessment should be approached with caution and combined with clear validation boundaries.

Reliability and quality control are also central to practical deployment. Recent work on test-time augmentation and quality-control procedures has shown that raw neural posterior probabilities may be insufficient for difficult regional phase-picking conditions [16]. Such methods improve reliability by evaluating prediction stability and filtering uncertain outputs. However, in an operational monitoring system, quality control must also interact with event declaration, phase-picking validity, physical consistency rules, inter-station agreement, and reduced-input behavior. Therefore, the present study does not claim that test-time augmentation or quality control is new by itself. Instead, it uses these concepts as part of a broader waveform-centered monitoring architecture that combines neural inference, graph-aware refinement, validation-only threshold selection, physical plausibility checks, and fallback operations when optional auxiliary branches are unavailable or rejected.

Rapid hazard-related characterization has also become an active direction in AI-assisted seismic monitoring. GRAPES demonstrated that real-time shaking information can be estimated from evolving seismic vectors without requiring all traditional source parameters as intermediate outputs [17]. GNSS-based and DAS-based approaches have also shown potential for rapid source or shaking characterization in cases where conventional seismic-only pipelines may be limited by saturation, geometry, or offshore source location [13,14,15]. Nevertheless, these approaches are often developed as separate downstream products rather than as components of a unified detection–picking–quality-control–characterization workflow. In the present study, the hazard-related head is therefore treated as an auxiliary rapid-characterization output, not as a substitute for full source inversion or comprehensive seismic hazard analysis.

Based on the reviewed literature, the unresolved problem can be formulated as follows: despite substantial progress in waveform-based AI detection, phase picking, graph-aware processing, and modality-specific sensing, there remains a need for a conservative and quality-controlled monitoring framework that preserves reliable event declaration and phase-picking behavior under variable waveform quality, incomplete auxiliary sensing, domain shift, and controlled noise degradation. This problem arises from the fragmentation of existing methods by task and sensor type, the scarcity of synchronized heterogeneous datasets, differences in sensor response and signal representation, and the vulnerability of AI predictions to unstable or physically inconsistent outputs. The present manuscript addresses this problem by developing and evaluating a waveform-centered, AI-assisted framework with optional auxiliary-sensor support, graph-aware refinement, post-inference quality control, and reduced-input fallback. Its empirical validation is intentionally limited to real waveform-centered IRIS records of the Almaty region, while optional DAS/MEMS/HR-GNSS branches are evaluated only as architectural extensions through controlled branch-availability and reduced-input scenarios.

## 3. The Aim and Objectives of the Study

The aim of this study is to develop and evaluate a quality-controlled, waveform-centered, AI-assisted framework for seismic event detection, P- and S-phase picking, graph-aware inter-station refinement, and auxiliary rapid hazard-related characterization under standard and degraded monitoring conditions. The framework is designed with optional DAS, MEMS, and high-rate GNSS branches; however, these branches are treated as auxiliary architectural extensions rather than as a fully validated, synchronized multimodal field deployment.

The primary empirical evaluation is based on real waveform-centered IRIS records from the Almaty seismic region. Therefore, the central validation question is whether the proposed monitoring architecture can improve the balance between event-detection accuracy, false-alarm control, detection latency, and phase-picking precision compared with conventional and AI-assisted waveform-based reference pipelines. The optional auxiliary branches are evaluated only through controlled branch-availability, reduced-input, fallback, and stress-test scenarios in order to assess architectural robustness under incomplete sensing conditions.

To achieve this aim, the following objectives were defined:

1. To analyze the methodological limitations of traditional seismic monitoring systems using a trigger-based workflow, with a focus on waveform-based AI, graph-based workflows for phase picking, and post-inference quality control;
2. To design a monitoring architecture to be waveform-centered, in which the seismic waveform branch is required and DAS, MEMS, and high-rate GNSS branches are optional auxiliary inputs that are only used after a reliability screening at the branch level;
3. To describe the model architecture, which encompasses the waveform encoder, event-detection head, P- and S-phase-picking heads, graph-aware refinement module, post-inference quality-control layer, and auxiliary magnitude-related rapid-characterization head;
4. To establish a train/validation/test partitioning scheme for validation, validation-only threshold selection, controlled C1–C3 noise conditions and reduced-input scenarios, and a single definition of false-alarm rate (FAR) for window normalization;
5. To compare the proposed complete monitoring pipeline with the conventional monitoring pipeline and with the PhaseNet-like and Earthquake Transformer-like pipelines using the precision, recall, F1 score, false-alarm rate, detection latency, and P/S phase-picking errors;
6. To assess the individual effect of the waveform backbone, graph-aware refinement, quality-control layer, optional auxiliary support, and reduced-input fallback with component-wise and ablation analysis;
7. To interpret the results conservatively in view of the limited number of independent test events; limited empirical validation scope focused on the waveform; and absence of a fully synchronized real field deployment of DAS, MEMS, and HR-GNSS and seismic sensors.

Accordingly, the study is structured not as a claim of universal superiority of a new neural predictor but as an evaluation of a complete monitoring-oriented framework. The comparison with baseline methods is interpreted as an operational pipeline comparison under the reported benchmark and controlled degradation conditions. This interpretation is necessary because the proposed framework includes not only waveform inference but also graph-aware refinement, quality-control rules, reduced-input logic, and optional auxiliary-branch handling.

## 4. Materials and Methods

### 4.1. Object of Research, Hypothesis and Assumptions

The object of this study is a waveform-centered, AI-assisted seismic monitoring framework designed for rapid event detection, P- and S-phase picking, graph-aware inter-station refinement, post-inference quality control, and auxiliary rapid hazard-related characterization. The mandatory input of the framework is the preprocessed seismic waveform branch. Optional DAS, MEMS, and high-rate GNSS branches are included only as auxiliary architectural extensions and are used only when synchronized, complete, and quality-controlled measurements are available. Therefore, in this study, the framework is not treated as a fully validated real-world DAS-MEMS-GNSS-seismic deployment but as a modular monitoring architecture whose primary empirical validation is based on waveform-centered seismic records.

**Figure 1:**
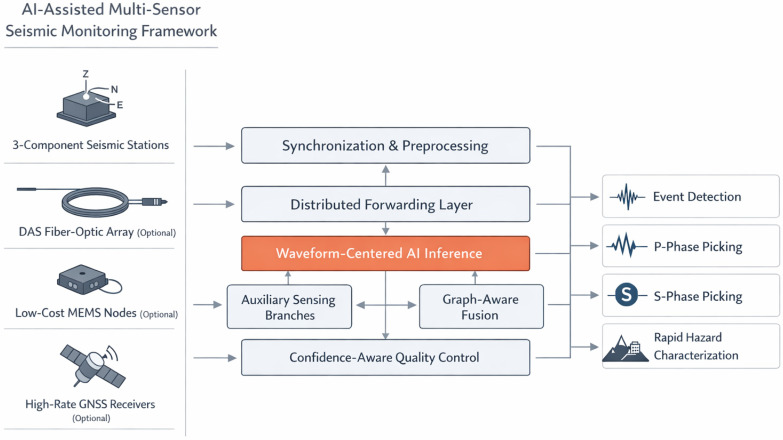
Structural architecture of the proposed AI-assisted multi-sensor seismic monitoring framework integrating waveform-centered seismic sensing, auxiliary DAS/MEMS/GNSS branches, synchronization and preprocessing, distributed inference support, graph-aware fusion, quality control, and rapid hazard-related outputs.

The proposed approach represents a distributed system architecture where preprocessed candidate waveform segments are acquired and forwarded close to the sensing nodes and higher-level decisions are made at the central analytics center. As a result, the current research framework aligns well with modern approaches to the development of energy-aware distributed edge AI systems capable of operating reliably in terms of communication and positioning constraints [18]. The structural architecture of the proposed multi-sensor seismic monitoring framework is shown in Figure 1.

As shown in Figure 1, the waveform branch is the mandatory path of the framework, whereas the DAS, MEMS, and high-rate GNSS branches are conditional auxiliary paths. If auxiliary measurements are unavailable, incomplete, unsynchronized, or rejected by branch-level quality control, the framework continues through the waveform-only fallback path. This design avoids treating optional auxiliary sensing as a prerequisite for inference and preserves consistency with the waveform-centered validation scope of the study.

One can formulate the hypothesis behind this research paper in the following manner: it is expected that the combination of waveform-oriented AI inference with confidence-based quality control, auxiliary sensing channels, and a modular distributed architecture would increase the efficiency and robustness of seismic monitoring under noisy conditions. It is expected that such a combination would deliver more stable results for event detection and more consistent and reliable P-wave and S-wave picking than the processing of individual modalities or individual events in isolation.

For methodological consistency, the following assumptions were adopted:

- The seismic waveform branch is the primary and mandatory source of information for event detection and P/S phase picking.
- DAS, MEMS, and high-rate GNSS observations are treated as optional auxiliary inputs rather than as mandatory empirical inputs.
- Auxiliary measurements are used only if they satisfy predefined requirements for time synchronization, signal completeness, amplitude validity, successful preprocessing, and branch-level reliability.
- Missing or rejected auxiliary branches do not invalidate the monitoring pipeline; in such cases, the framework operates in waveform-only fallback mode.
- Event and phase labels are obtained from catalog-based or verified benchmark annotations.
- The framework is intended for rapid monitoring-oriented inference, not for full source inversion, detailed rupture modeling, or comprehensive post-event hazard analysis.
- Different sensing modalities are not forced into a physically misleading common raw waveform format; instead, they are processed through modality-specific preprocessing and combined only at later decision or feature-fusion stages.

The study also relies on several simplifications that define the boundary of interpretation. First, the empirical validation of the primary monitoring core is based on waveform-centered seismic records, whereas optional DAS/MEMS/HR-GNSS branches are evaluated through controlled architectural scenarios. Second, the framework does not model full seismic wave propagation, hypocentral inversion, rupture dynamics, or site-response effects. Third, controlled noise and reduced-input experiments are used as reproducible stress tests and should not be interpreted as complete physical reproductions of all real seismic noise or all real multi-sensor field conditions. These simplifications are necessary to keep the validation protocol reproducible and to avoid overclaiming beyond the available dataset.

The functional decomposition of the framework is summarized in Table 1. The table separates the mandatory waveform path, optional auxiliary sensing, preprocessing, distributed forwarding, waveform inference, graph-aware refinement, quality control, and hazard-related output generation. This decomposition is used to define the methodological role of each component before the signal representation and model architecture are described in the following subsections.

As shown in Table 1, the proposed framework is organized as a modular monitoring architecture rather than as a rigid, permanently multimodal system. The waveform inference backbone remains the central processing path, while auxiliary branches are conditional components that may support robustness when valid measurements are available. This decomposition also clarifies the role of later ablation experiments, where the waveform backbone, graph-aware refinement, quality-control layer, and full framework are evaluated separately.

### 4.2. Signal Representation and Preprocessing Model

The signal representation and preprocessing model was developed to support waveform-centered seismic monitoring and to use additional acquisition branches when reliable synchronized data is available. The most important empirical element of this study is the seismic waveform branch, which is measured at a frequency of 100 Hz and is extracted as a 240-s analytical window focused on the catalog-reported P-wave arrival. Additional high-rate DAS, MEMS, and GNSS data are treated as auxiliary inputs and are not required for the primary waveform-centered empirical benchmark. Thus, the preprocessing model separates the mandatory preparation of the waveform from the conditional transformation of the auxiliary branch.

Let $x_{w}(t)$ denote the mandatory seismic waveform input, where w refers to the waveform branch. For the reported Almaty-region benchmark, $x_{w}(t)$ corresponds to a single vertical-component HHZ trace. When three-component records are available, the waveform input may be extended to $x_{w}(t)=[x_{Z}(t),x_{N}(t),x_{E}(t)]$, but this extension was not the basis of the reported empirical benchmark. For an analysis window of length L, the waveform-centered input segment can be represented as

(1) $$X_{w}={\left\{x_{w}(t_{i})\right\}}_{i=1}^{N},N=Lf_{s},$$

where $f_{s}=100$ Hz in the reported benchmark and L=240 s.

For optional auxiliary modality m, where m∈{DAS,MEMS,HR-GNSS}, the corresponding input is denoted as $x_{m}(t)$. These auxiliary observables differ physically from conventional seismic waveforms. DAS measurements represent fiber-coupled strain or strain-rate responses along a spatially distributed cable; MEMS sensors provide acceleration-related measurements with device- and installation-dependent noise characteristics, and high-rate GNSS provides displacement-related information that may be useful mainly for larger events. Consequently, these inputs are not converted into a single physically equivalent raw waveform format.

The synchronized multi-branch observation can be written in a general form as

(2) $$X\left(t\right)=\{X_{w},X_{m}|m\in M_{aux}\},$$

where $X_{w}$ is the mandatory waveform segment and $M_{aux}$ is the set of available auxiliary modalities. If no auxiliary modality is available or if an auxiliary branch fails quality control, set $M_{aux}$ is empty, and the framework operates in waveform-only mode. This formulation preserves the mandatory waveform path while allowing for conditional auxiliary support.

Before inference, each waveform segment underwent demeaning, detrending, optional tapering, band-limited filtering, amplitude normalization, resampling where applicable, and fixed-window extraction. In the reported waveform-centered benchmark, the sampling rate was fixed at 100 Hz, and each input window had a duration of 240 s. In the reported waveform-centered benchmark, the sampling rate was fixed at 100 Hz, each input window had a duration of 240 s, and a zero-phase fourth-order Butterworth band-pass filter with an effective passband of 1.0–45.0 Hz was applied before normalization and model-input construction. These preprocessing steps follow common waveform-centered deep learning practice and are compatible with the reproducible benchmarking logic used in SeisBench-ready workflows [19].

To preserve modality-specific information, each branch is transformed by its own preprocessing and feature-construction operator:

(3) $$Z_{m}=\Phi_{m}(X_{m}),$$

where $\Phi_{m}(\cdot )$ denotes the transformation assigned to modality m. For the seismic waveform branch, $\Phi_{w}(\cdot )$ includes waveform normalization and temporal feature encoding. For DAS, $\Phi_{DAS}(\cdot )$ should preserve the spatial–temporal panel structure rather than reducing the signal to a single-point waveform. For MEMS, $\Phi_{MEMS}(\cdot )$ should account for acceleration scaling and branch-level reliability. For high-rate GNSS, $\Phi_{GNSS}(\cdot )$ should account for displacement- or velocity-related features. This branch-specific formulation is consistent with recent work showing that GNSS and other auxiliary observables require representation models different from ordinary seismic waveforms [20].

The model-ready representation is then formed as

(4) $$Z=F(Z_{w},{\left\{Z_{m}\right\}}_{m\in M_{valid}}),$$

where $Z_{w}$ is the waveform representation, and $M_{valid}$ is the subset of auxiliary branches that passed branch-level quality control. The F(⋅) operator denotes either feature-level or decision-level construction, depending on the experimental configuration. In the primary empirical benchmark, F(⋅) reduces to the waveform-centered representation because synchronized DAS/MEMS/HR-GNSS data were not used as a fully empirical multimodal deployment. In optional-branch experiments, F(⋅) is used only after auxiliary inputs satisfy synchronization, completeness, amplitude-validity, and preprocessing-success criteria.

**Figure 2:**
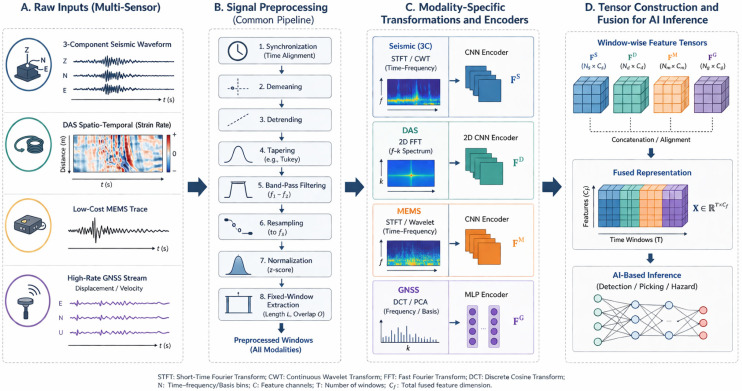
Signal preprocessing and multi-branch representation workflow of the proposed seismic monitoring framework, including synchronization, normalization, band-limited filtering, fixed-window extraction, modality-specific transformation, and tensor construction for subsequent AI-based inference: (**A**) Raw Input (Multi-Sensor); (**B**) Signal Preprocessing (Common Pipeline); (**C**) Modality-Specific Transformations and Encoders; (**D**) Tensor Construction and Fusion for AI inference.

The preprocessing and branch-representation workflow is summarized in Figure 2. Figure 2 distinguishes the mandatory waveform path from dashed optional auxiliary branches and shows that auxiliary inputs enter the framework only after branch-specific preprocessing and quality-control screening.

As shown in Figure 2, the preprocessing step does not force heterogeneous inputs into a common raw format for heterogeneous detection methods. Instead, each branch is transformed based on its own signal structure, and only valid branch representations are transmitted at later stages of merging or decision making. This design corresponds to the task of validating research, in which waveform-centered empirical validation is fundamental, and auxiliary sensing is valued as additional architectural support.

The principal preprocessing and representation parameters are summarized in Table 2. These parameters define the waveform-centered benchmark and identify which auxiliary-branch parameters must be specified when synchronized auxiliary data become available.

As summarized in Table 2, only the waveform parameters are fixed for the reported empirical benchmark. Auxiliary-branch parameters are included to define the architecture but do not imply that a fully synchronized DAS/MEMS/HR-GNSS/seismic-field dataset was used in the primary validation. This distinction is essential for interpreting the subsequent model architecture and experimental protocol.

The resulting representation model provides a consistent input basis for the proposed inference framework while preserving the physical differences among sensing modalities. In the following subsection, this representation is connected to the model architecture, including the waveform encoder, task-specific output heads, graph-aware refinement module, quality-control layer, and auxiliary hazard-related head.

### 4.3. Model Architecture, Training Configuration, and Multi-Task Inference

The proposed model was designed as a modular monitoring-oriented pipeline rather than as a new stand-alone phase picker. Its purpose was to combine waveform-based event detection, P- and S-phase picking, graph-aware station-consistency refinement, auxiliary magnitude-related rapid characterization, and post-inference quality control within a single operational workflow. Accordingly, the contribution of the model is evaluated at the pipeline level and not as a claim of intrinsic superiority over established waveform-based neural architectures such as PhaseNet-like or Earthquake Transformer-like models.

The mandatory model input is the preprocessed waveform representation. In the reported Almaty-region benchmark, this input corresponds to a single vertical HHZ waveform channel sampled at 100 Hz and extracted as a 240 s P-centered analysis window. If three-component waveform records are available, the same architecture can be extended to three input channels; however, this extension was not the basis of the reported empirical benchmark. Optional DAS, MEMS, and high-rate GNSS representations are treated as conditional auxiliary inputs and are permitted to contribute only after branch-level synchronization, completeness, amplitude-validity, preprocessing-success, and reliability checks.

The waveform branch uses a compact, one-dimensional encoder–decoder architecture. The encoder consists of five Conv1D blocks with batch normalization and ReLU activation. The number of channels increases from 16 to 256, while the kernel size decreases from 7 to 3 across deeper blocks. The decoder uses upsampling and Conv1D operations to reconstruct a sample-wise temporal representation for P- and S-phase probability estimation. The architecture was intentionally kept compact to reduce overfitting risk on the limited regional dataset and to support low-latency monitoring-oriented inference.

**Figure 3:**
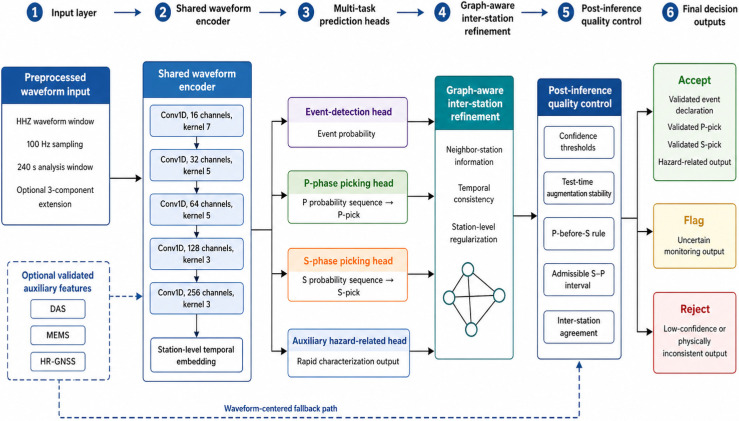
Operational architecture of the proposed multi-task inference model, including waveform-centered input representation, shared waveform encoder, task-specific event-detection and phase-picking heads, graph-aware inter-station refinement, auxiliary hazard-related output, and post-inference quality-control logic.

As shown in Figure 3, the predictive backbone and the post-inference quality-control layer are intentionally separated. The neural model produces event, phase, and auxiliary hazard-related outputs, whereas the quality-control layer evaluates confidence, stability, physical plausibility, and branch or station agreement before final monitoring decisions are accepted, flagged, or rejected. This separation is important because later ablation experiments evaluate the waveform backbone, graph-aware refinement, quality-control layer, and full framework as distinct components.

The detailed waveform-backbone configuration is summarized in Table 3. The table reports the exact number of input channels, convolutional channels, kernel sizes, and output roles used in the architecture.

The detailed configuration of the waveform inference backbone is summarized in Table 3.

As shown in Table 3, the backbone was intentionally kept compact to reduce overfitting risk on the limited regional dataset and to support low-latency monitoring. The encoder blocks extracted progressively higher-level temporal waveform features, while the decoder reconstructed sample-wise representations required for P- and S-phase probability estimation.

The event-detection head maps the shared waveform representation ($H_{w}$) to an event posterior probability:

(5) $$p_{det}=\sigma (g_{det}(H_{w})),$$

where $g_{det}(\cdot )$ is the detection head and σ(⋅) is the sigmoid activation function. A binary event declaration is produced only after applying the validation-selected decision threshold and the post-inference quality-control rules described in Section 4.4.

The P- and S-phase picking heads produce sample-wise posterior probability sequences:

(6) $$p_{P}=g_{P}\left(H_{w}\right),p_{S}=g_{S}(H_{w}),$$

where $P_{P}$ and $P_{S}$ denote the posterior probability sequences for P- and S-arrivals. The estimated arrival times were selected as the time samples with maximum posterior probability:

(7) $${\hat{t}}_{P}=arg\underset{t}{\max}p_{P}(t),{\hat{t}}_{S}=arg\underset{t}{\max}p_{S}(t),$$

These estimates are considered valid only if they satisfy the confidence, stability, and physical-consistency criteria defined in the quality-control layer.

The auxiliary hazard-related head is connected after the shared representation and quality-controlled event-declaration pathway. Depending on the available label definition, it may be formulated either as a regression head for a continuous hazard-related indicator or as a classification head for categorical severity levels. In this study, this output is interpreted as an auxiliary rapid-characterization component and not as a substitute for full source inversion, rupture modeling, or comprehensive seismic hazard analysis. In this study, the hazard-related target was implemented as a three-class catalog-magnitude severity proxy derived from event metadata: low magnitude-related severity (M < 3.0), moderate magnitude-related severity (3.0 ≤ M < 4.0), and higher regional magnitude-related severity (M ≥ 4.0). Events without reliable catalog magnitude metadata were excluded from this auxiliary head evaluation. This target was used only for rapid preliminary characterization and was not treated as a prediction of PGA, PGV, macroseismic intensity, structural damage, or full seismic hazard.

Graph-aware refinement is introduced to improve station-level temporal consistency after local waveform inference. Let $h_{i}$ denote the embedding or prediction state associated with station i, and let N(i) denote the set of neighboring stations connected to station i. The refined state can be written as

(8) $$h_{i}^{\prime }=\Psi (h_{i},\left\{h_{j}:j\in N(i)\right\}),$$

where Ψ(⋅) is the graph-based refinement function. In this study, the graph module is used as a consistency-refinement layer rather than as an independent event detector. The station graph was defined as a fully connected, distance-weighted graph over the available seismic stations for each event window. Each node represents one station-level waveform prediction state, and each edge represents the spatial relationship between two stations. The edge weight between stations i and j was computed from the inter-station distance (d_ij_) as $w_{ij}=exp(-\frac{d_{ij}}{d_{0}})$, where $d_{0}$ is the median non-zero inter-station distance within the seven-station network. Therefore, the graph module was used as a station-consistency refinement layer rather than as an independent physical propagation or event-location model.

Optional auxiliary branches are used only after branch-level validation. If valid high-rate DAS, MEMS or GNSS representations are available, their transformed features may contribute to late fusion or hazard-related auxiliary characterization. If one or more secondary branch is unavailable, incomplete, unsynchronized or rejected, the model will continue to work only with the waveform. This reduced-input operation logic is part of the framework’s concept and is evaluated separately in experiments with reduced-input operation and branch availability.

The complete multi-task objective combines detection, phase-picking, and auxiliary hazard-related terms:

(9) $$L=\lambda_{det}L_{det}+\lambda_{pick}L_{pick}+\lambda_{haz}L_{haz},$$

The detection loss is implemented as a binary cross entropy for event declaration. The phase-picking loss is based on the time error of the arrival of P and S or the loss of phase probability in the sample, depending on the label representation. The term hazard-related is used as the average absolute error for continuous purposes or as the cross entropy for categorical purposes. The hazard-related target definition should correspond to the target definition mentioned above.

The loss weights used in the reported training configuration are summarized in Table 4. These weights were fixed before final test evaluation and were not tuned on the test subset.

As shown in Table 4, the phase-picking term was assigned the largest weight because arrival-time precision is critical for downstream seismic monitoring tasks. The hazard-related loss was assigned a smaller weight because this branch was treated as an auxiliary output rather than as the primary optimization target.

The training configuration used for the learning-based models is summarized in Table 5.

As shown in Table 5, model selection and threshold tuning were performed using the validation subset, while the test subset was reserved for final reporting. The reported results correspond to a fixed event-level split; multiple-seed, repeated-run statistics are not claimed in the present revision.

For objective interpretation, the comparison between M1, M2, M3, and M4 is treated as an operational pipeline comparison. M1 is a conventional trigger-based workflow; M2 is a PhaseNet-like, waveform-centered AI baseline; M3 is an Earthquake Transformer-like joint detection-and-picking baseline; and M4 is the proposed complete monitoring pipeline with waveform inference, graph-aware refinement, post-inference quality control, reduced-input operation, and optional auxiliary-branch handling. Therefore, performance differences should not be interpreted as evidence that the M4 waveform backbone, itself, is superior to all existing neural architectures. Instead, they indicate whether the complete monitoring pipeline improves performance under the specified validation conditions.

The architecture described in this subsection defines the predictive part of the proposed framework. The post-inference quality-control layer, including reliability thresholds, augmentation-stability analysis, physical consistency checks, and accept/flag/reject decision logic, is described in Section 4.4.

### 4.4. Quality Control and Uncertainty Handling

The quality-control layer was introduced after neural inference in order to convert raw probabilistic outputs into operational monitoring decisions. It is not a separate predictive model and does not replace the waveform encoder, phase-picking heads, or graph-aware refinement module. Its function is to evaluate whether event declarations and phase picks are sufficiently reliable, stable, and physically plausible before they are passed to final monitoring output or auxiliary hazard-related characterization.

The term “physically implausible outputs” refers to model predictions that violate basic seismological or acquisition-related constraints after inference. Such outputs may occur under a low SNR, waveform distortion, incomplete station coverage, domain shift, or unstable neural posterior probabilities. Examples include an S-pick predicted before a P-pick, a phase pick outside the analysis window, an S–P interval outside the admissible regional range, and mutually inconsistent event declarations across stations or valid branches. These outputs are not interpreted as physically impossible seismic signals; rather, they are treated as unreliable model outputs that should be filtered, flagged, or rejected before final monitoring decisions are produced.

For event detection, a generic confidence score is defined as

(10) $$q_{det}={\hat{y}}_{det},$$

where ${\hat{y}}_{det}$ is the posterior event probability defined in Equation (6). A predicted event is accepted only if

(11) $$q_{det}\geq \tau_{q,det},$$

where $\tau_{q,det}$ is the confidence threshold estimated from the validation set.

For phase picking, the confidence of the P and S predictions is defined as the maximum posterior probability over the corresponding sequence:

(12) $$q_{P}\underset{t}{\max}{Ρ}^{P}\left(t\right),q_{S}=\underset{t}{\max}{Ρ}^{S}(t),$$

A P-pick or S-pick is accepted only if

(13) $$q_{P}\geq \tau_{q,P},q_{S}\geq \tau_{q,S},$$

where $\tau_{q,P}$ and $\tau_{q,S}$ are validation-derived phase-confidence thresholds.

In addition to posterior confidence, the proposed framework imposes physical consistency rules. First, a valid pair of arrivals must satisfy the following basic temporal ordering:

(14) $$0<{\hat{t}}_{P}<{\hat{t}}_{S}<L,$$

where L is the analysis-window length. Second, the estimated S–P interval must remain within an admissible range determined by the target acquisition geometry and monitoring context:

(15) $${∆}_{min}<{\hat{t}}_{S}-{\hat{t}}_{P}<{∆}_{max},$$

These rules are not intended as substitutes for travel-time inversion or precise hypocentral constraints; rather, they act as practical filters for rejecting physically implausible outputs before they propagate to later monitoring stages.

Prediction stability was assessed using augmentation-based consistency analysis. Let ${\left\{{\hat{t}}_{P}^{\left(a\right)}\right\}}_{a=1}^{A}$ denote the set of P-pick estimates obtained from augmented versions of the same input window (A). The P-pick dispersion is defined as

(16) $$\sigma_{P}\sqrt{\frac{1}{A}\sum_{a=1}^{A}{\left({\hat{t}}_{P}^{a}-{\bar{t}}_{p}\right)}^{2}},$$

where ${\bar{t}}_{P}$ is the mean augmented estimate. Analogously, for S-picks,

(17) $$\sigma_{S}\sqrt{\frac{1}{A}\sum_{a=1}^{A}{\left({\hat{t}}_{S}^{a}-{\bar{t}}_{S}\right)}^{2}}.$$

A pick is considered augmentation-stable only if

(18) $$\sigma_{P}\leq \tau_{\sigma ,P},\sigma_{S}\leq \tau_{\sigma ,S},$$

where $\tau_{\sigma ,P}$ and $\tau_{\sigma ,S}$ are admissible dispersion thresholds.

When multiple stations or valid auxiliary branches are available, an agreement score is used to evaluate consistency across contributing sources. Let $u_{k}$ denote the normalized confidence contribution from station or valid branch k, where k=1,…,K. A simple agreement score can be written as

(19) $$Q_{agg}=\frac{1}{K}\sum_{k=1}^{K}u_{k},$$

Outputs with $Q_{agg}$ below the validation-selected threshold are not automatically treated as correct or incorrect. Instead, they are flagged as reduced-confidence outputs. This rule is important because disagreement may reflect either poor signal quality or genuine incomplete sensing, especially when optional auxiliary branches are unavailable or rejected.

The quality-control thresholds were selected only on the validation subset and were fixed before final test evaluation. The threshold values used in the revised validation protocol are summarized in Table 6.

As shown in Table 6, the threshold selection procedure was separated from the final test. This was necessary to avoid an optimistic bias, since the degree of quality control directly affects the frequency of false-positive results, the frequency of correct estimates, and the number of accepted or noted forecasts. Thus, the validation subset was used to determine all quality-control thresholds, while the test subset was used only to evaluate the fixed decision rules.

The final quality-control decision has three possible outcomes: accept, flag, or reject. An output is accepted when the confidence thresholds, stability thresholds, and physical consistency rules are satisfied. An output is flagged when the mandatory physical rules are satisfied but confidence, stability, or agreement is marginal. A flagged output can still be reported as a reduced-confidence monitoring result, but it is not treated as a fully reliable detection or pick. An output is rejected when it violates mandatory physical rules, fails minimum confidence requirements, or comes from an auxiliary branch that fails branch-level quality control.

**Figure 4:**
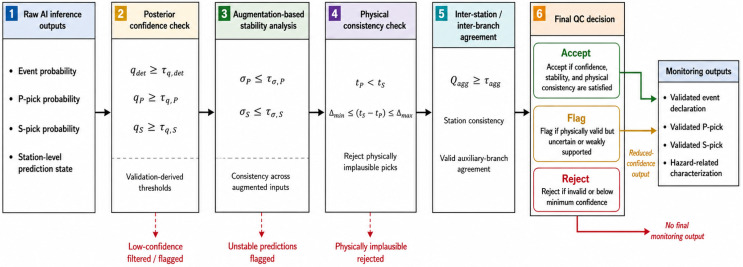
Quality-control and uncertainty-handling workflow, including posterior confidence estimation, augmentation-based stability analysis, physical consistency checks, inter-station or inter-branch agreement assessment, and final accept/flag/reject decision logic.

The workflow of the post-inference quality-control layer is shown in Figure 4. The figure presents the decision sequence from raw neural outputs to confidence assessment, augmentation-stability analysis, physical consistency checking, station or branch agreement assessment, and final accept/flag/reject output.

As shown in Figure 4, the quality-control layer acts after the predictive model and before final generation of the monitoring output. This design prevents unstable detections, low-confidence picks, and physically inconsistent predictions from being propagated directly to downstream event declaration or hazard-related characterization. Thus, the QC layer provides operational reliability control while keeping the neural inference model and the post-inference decision logic analytically separable.

The quality-control procedure also defines how uncertainty is represented in the framework. Accepted outputs are treated as valid monitoring results under the adopted validation protocol. Flagged outputs indicate reduced confidence and should be considered separately in downstream operational use. Rejected outputs are excluded from final performance scoring, except where the rejection rate or valid pick rate is explicitly reported. Therefore, Section 5 reports not only accuracy-related metrics but also the number or proportion of accepted, flagged, and rejected outputs where applicable.

The quality-control logic described in this subsection is applied consistently across the standard benchmark, controlled noise conditions, reduced-input scenarios, and ablation experiments. Because threshold selection is performed only on the validation subset, the final test results reflect the behavior of fixed decision rules rather than post hoc tuning. The next subsection describes the experimental configuration and validation protocol used to evaluate these fixed rules, together with the waveform-centered inference model.

### 4.5. Experimental Configuration, Validation Protocol, and Statistical Analysis

#### 4.5.1. Validation Scope and Dataset Description

The experimental evaluation was designed as a hybrid validation protocol. The primary empirical component of the study was based on real waveform-centered seismic records, whereas controlled perturbation, reduced-input, branch-availability, and ablation experiments were used to evaluate the robustness and operational behavior of the proposed framework under degraded or incomplete sensing conditions. This distinction was introduced to avoid mixing direct real-data validation with controlled architectural stress testing.

The real-data benchmark was constructed from waveform records obtained through FDSN-compatible IRIS/EarthScope data services for the Almaty seismic region. The reference point was Almaty city, Kazakhstan (43.22° N, 76.92° E), and the spatial selection radius was 3.0° (approximately 333 km). The analyzed corpus included seven seismic stations available through regional Central Asian FDSN networks: TARG, ARLS, BAET, JNKS, BTLS, SHLS, and TLG. The observation period extended from 1 January 2023 to 31 December 2024. Only vertical HHZ records sampled at 100 Hz were used in the primary empirical benchmark. In total, the waveform-centered corpus contained 219 seismic events and 1260 event traces. Each event trace was extracted as a 240 s analysis window, including 120 s before and 120 s after the reported P-wave arrival. These dataset characteristics are summarized in Table 7.

As shown in Table 7, the primary empirical benchmark is based on real waveform-centered seismic records. Synchronized DAS, MEMS, and high-rate GNSS data were not used as a complete real-world multimodal deployment in this validation. Therefore, the reported comparative metrics for M1–M4 primarily reflect the behavior of the waveform-centered monitoring core under real Almaty-region records and controlled degradation scenarios.

The station metadata used to document the regional waveform corpus are summarized in Table 8. The table includes the station codes, network affiliations, available geographic coordinates, and operational notes for the benchmark stations used in this study.

**Figure 5:**
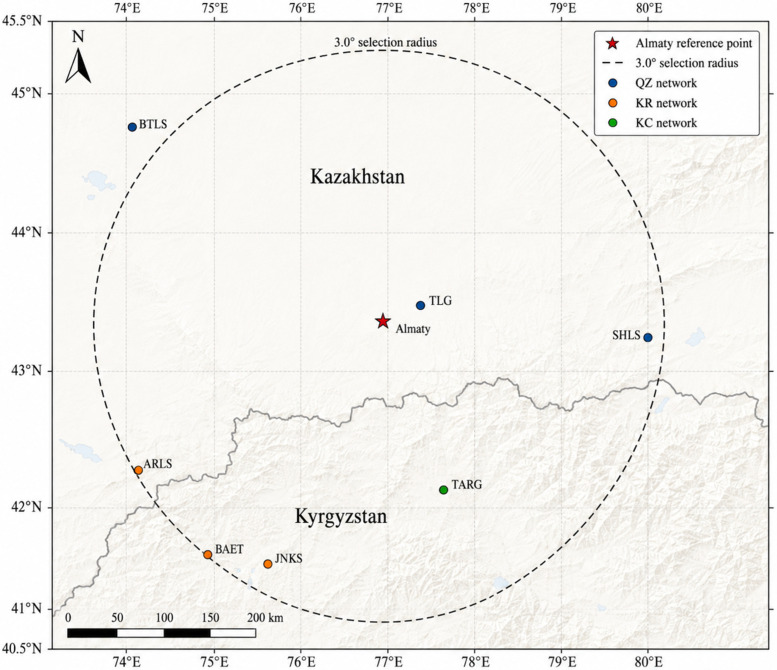
Study area and station distribution for the Almaty-region waveform-centered benchmark. The map shows the Almaty reference point, the 3.0° selection radius, and the seven seismic stations used in the empirical HHZ waveform corpus. Station colors indicate FDSN network affiliation: QZ, KR, and KC.

The station geometry and FDSN network affiliation of the waveform-centered Almaty-region benchmark are shown in Figure 5.

To improve reproducibility, the waveform corpus was defined using FDSN-compatible data-access criteria. Station metadata, event metadata, waveform time series, and response information were obtained or verified through standard FDSN services, including station metadata, waveform time-series retrieval, event-parameter queries, and data-availability checks. The selected stations belong to regional Central Asian networks indexed by FDSN: QZ for the SEME network in Kazakhstan, KR for the Kyrgyz Digital Network, and KC for the Central Asian Seismic Network of CAIAG. Published regional station tables confirm the coordinates for TLG, BTLS, SHLS, ARLS, JNKS, and TARG. BAET was retained in the corpus because it is available through the KR network and is listed in FDSN station metadata with a latitude of 41.0899° N, longitude of 75.0148° E, elevation of 3015 m, and start date of 9 July 2024. This shorter operational period explains the lower availability of BAET records during the 2023–2024 interval and is consistent with recent Almaty-region waveform studies. These metadata were used only to document data provenance and station-network affiliation; the empirical benchmark, itself, remained waveform-centered and used HHZ traces sampled at 100 Hz.

#### 4.5.2. Event-Level Data Partitioning

To prevent information leakage, the dataset was divided at the event level rather than at the trace level. All traces associated with the same seismic event were assigned to the same subset. This procedure was necessary because a single earthquake may be recorded by several stations; if traces from the same event were distributed across training and test subsets, the final evaluation could be optimistically biased.

The dataset was divided into training, validation, and test subsets using a 70/15/15 event-level split. Of the 219 seismic events, 153 events were assigned to the training subset, 33 events to the validation subset, and 33 events to the test subset. The training subset was used for model fitting. The validation subset was used for hyperparameter selection, model selection, decision-threshold tuning, and quality-control threshold selection. The test subset was held out and used only for final performance evaluation.

The event-level partitioning scheme is summarized in Table 9. The approximate number of traces reflects proportional assignment of the 1260 event traces after event-level grouping. Because all traces from the same earthquake were kept within the same subset, the split is interpreted primarily by the number of independent events rather than by approximate trace counts.

As shown in Table 9, the final test subset contains a limited number of independent seismic events. Therefore, the reported performance differences must be interpreted cautiously and should be accompanied by uncertainty estimates, repeated-run statistics, and paired comparisons where possible.

#### 4.5.3. Benchmark Methods and Evaluation Configurations

Four monitoring configurations were evaluated. The comparison was organized as an operational pipeline comparison rather than as a direct claim that one neural backbone is intrinsically superior to another. This distinction is important because the proposed framework includes waveform inference, graph-aware refinement, post-inference quality control, reduced-input fallback, and optional auxiliary-branch handling.

The compared configurations are summarized in Table 10. M1 represents a conventional trigger-based workflow, M2 represents a PhaseNet-like waveform-centered AI baseline, M3 represents an Earthquake Transformer-like joint detection-and-picking baseline, and M4 corresponds to the proposed complete framework.

As shown in Table 10, the compared methods represent different levels of operational complexity. M1 is a standard monitoring system that is not based on deep learning; M2 and M3 provide basic AI-assisted, waveform-oriented data; and M4 is the proposed comprehensive monitoring pipeline. Therefore, this comparison should not be interpreted as a statement that the M4 predictive base, itself, is fundamentally superior to existing neural architectures. Instead, the comparison is used to assess whether the entire monitoring pipeline, including refinement and quality-control mechanisms, improves the behavior of the monitoring system under certain validation conditions.

To avoid an asymmetric interpretation of the comparison, the evaluated methods are not presented as identical neural backbones. M2 and M3 represent waveform-based AI reference workflows, whereas M4 represents a complete monitoring pipeline that includes waveform inference, graph-aware refinement, post-inference quality control, reduced-input fallback, and optional auxiliary-branch handling. Therefore, the purpose of the comparison is to evaluate operational monitoring behavior under a common validation protocol, not to prove that the M4 waveform encoder, alone, is superior to PhaseNet-like or Earthquake Transformer-like architectures. The ablation study in Section 5.4 was included specifically to separate the effects of the waveform backbone, graph-aware refinement, quality control, and the complete framework.

To make this distinction clearer, the contribution of individual M4 components was further investigated using ablative analysis. During this analysis, the signal base, graphics processing, quality-control level, and complete structure were separately evaluated. This allowed the study to distinguish the contribution of the predictive base from the contribution of reliability mechanisms post inference. The same evaluation metrics were applied to all configurations to ensure consistent reports on detection accuracy, false-alarm rate, delays, and phase-picking errors.

The test configurations defined in this subsection were used under both standard waveform conditions and controlled decomposition conditions. The following subsection describes conditions C1, C2, and C3, which were used for reliability testing.

#### 4.5.4. Controlled Noise Conditions (C1–C3)

Robustness was evaluated using three controlled evaluation conditions, denoted as C1, C2, and C3. These conditions were applied to the same event-level test subset described in Section 4.5.2. Therefore, differences in performance across C1–C3 reflected the effect of controlled signal degradation rather than changes in the composition of the test events.

C1 corresponds to the standard evaluation condition and uses the original preprocessed waveform test windows without additional perturbation. C2 corresponds to a controlled medium-noise stress-test condition in which band-limited perturbation is added to obtain a target SNR range of 10–15 dB. C3 corresponds to a stronger low-SNR stress-test condition with a target SNR range of 0–5 dB. When event-free empirical noise intervals are available, they are preferred as perturbation sources; otherwise, band-limited noise within the preprocessing passband is used to maintain a reproducible degradation protocol. These conditions are intended for controlled robustness comparison only and are not presented as complete physical models of natural seismic noise.

The perturbed waveform for condition k was defined as

(20) $$x_{k}\left(t\right)=x\left(t\right)+\alpha_{K}n_{b}\left(t\right),$$

where x(t) is the original preprocessed waveform, n_b_(t) is a band-limited noise sequence, and α_k_ is a scaling coefficient selected to obtain the target SNR range for condition k. The scaling coefficient was computed as

(21) $$\alpha_{k}=\sqrt{\frac{P_{x}}{P_{n}{\times 10}^{\frac{{SNR}_{k}}{10}}}},$$

where $P_{x}$ is the power of the original waveform segment, $P_{n}$ is the power of the unscaled noise sequence, and $SNR_{k}$ is the target signal-to-noise ratio expressed in decibels. The controlled evaluation conditions are summarized in Table 11.

As shown in Table 11, C2 and C3 should be interpreted as reproducible stress-test conditions. They do not fully reproduce real seismic noise, which may be colored, nonstationary, site-dependent, source-dependent, and affected by anthropogenic or environmental processes. Therefore, robustness results under C1–C3 are used for controlled comparison only and do not replace broader validation under natural low-SNR field conditions.

#### 4.5.5. Reduced-Input and Optional-Branch Evaluation

To assess whether this architecture remains functional in the event of the unavailability, incompleteness, unsynchronized timing, or rejection of auxiliary sensing branches during quality control at the branch level, experiments were conducted with a reduced-input configuration. In all scenarios, the waveform branch remained mandatory. The DAS, MEMS, and high-rate GNSS branches were considered as additional auxiliary inputs and were used only if the criteria of synchronization, completeness, amplitude validity, preprocessing success, and reliability were met at the branch level.

The reduced-input and optional-branch evaluation scenarios are summarized in Table 12.

As shown in Table 12, these scenarios evaluate the fault tolerance and reliability of an architecture with a reduced-input configuration. They should not be interpreted as empirical validation of the simultaneous integration of DAS, MEMS, high-rate GNSS, and seismic waveform data. To fully confirm such an implementation scheme, synchronized field data from all relevant sensing modalities is required.

#### 4.5.6. Evaluation Metrics and False-Alarm Rate Definition

The quantitative evaluation was based on event-detection accuracy, false-alarm control, detection latency, phase-picking accuracy, and auxiliary hazard-related performance where applicable. The same metric definitions were used for all compared configurations and all evaluation conditions.

For event detection, true positives (TPs) are correctly declared event windows, false positives (FPs) are event declarations in noise-only windows, and false negatives (FNs) are missed event windows. Precision is defined as

(22) $$Precision=\frac{TP}{TP+FP},$$

recall is defined as

(23) $$Recall=\frac{TP}{TP+FN},$$

and the F1 score is given by

(24) $$F1=\frac{2\times Precision\times Recall}{Precision+Recall}.$$

In the revised protocol, the false-alarm rate was defined using a single window-normalized computational standard. Specifically, FAR was computed as the number of false event declarations divided by the number of evaluated noise-only windows:

(25) $$FAR=\frac{N_{FP}}{N_{noise}},$$

where $N_{FP}$ is the number of false event declarations and $N_{noise}$ is the number of evaluated noise-only windows. Therefore, in this study, FAR is interpreted as the probability of producing a false event declaration per evaluated noise-only window, not as a rate per unit time.

Detection latency is defined as

(26) $$T_{lat}={\hat{t}}_{det}-t_{det}^{ref},$$

where ${\hat{t}}_{det}$ is the estimated detection time and $t_{det}^{ref}$ is the reference detection time. In the reported tables, detection latency is expressed in milliseconds and summarized across the test subset.

For phase picking, the absolute P- and S-arrival errors were computed as

(27) $$e_{P}=\left|{\hat{t}}_{P}-t_{P}\right|,$$

(28) $$e_{S}=\left|{\hat{t}}_{S}-t_{S}\right|,$$

where ${\hat{t}}_{P}$ and ${\hat{t}}_{S}$ are the estimated P- and S-arrival times and $t_{P}$ and $t_{S}$ are the corresponding reference arrival times. The main reported phase-picking metric is the mean absolute error for P- and S-picks. Where applicable, the median absolute error and valid-pick rate were also considered to distinguish timing accuracy from the number of accepted predictions.

For the hazard-related output, the metric depended on the formulation of the target. If the hazard-related head was treated as a regression output, the mean absolute error was computed as

(29) $${MAE}_{haz}=\frac{1}{N}\sum_{i=1}^{N}\left|{\hat{y}}_{i}-y_{i}\right|.$$

If the hazard-related output is categorical, the macro-F1 is used. The exact target definition and label source should be stated explicitly, as in this study, the hazard-related target was defined as an auxiliary catalog-magnitude severity proxy derived from the event magnitude reported in the event metadata. It was not treated as a direct prediction of PGA, PGV, macroseismic intensity, structural damage, or full seismic hazard. The auxiliary target was grouped into three classes: low magnitude-related severity (M < 3.0), moderate magnitude-related severity (3.0 ≤ M < 4.0), and higher regional magnitude-related severity (M ≥ 4.0). Events without a reliable catalog magnitude were excluded from the auxiliary hazard-head evaluation. Therefore, the reported hazard-related macro-F1 should be interpreted only as a preliminary rapid characterization result, not as validation of a complete seismic hazard assessment model.

The principal metrics used in the validation protocol are summarized in Table 13.

No composite “overall trade-off” score was used in the revised validation protocol. Instead, the comparison was based on separate quantitative metrics: precision, recall, F1 score, FAR, detection latency, P-pick MAE, S-pick MAE, and auxiliary hazard-related macro-F1 where applicable. Therefore, the phrase “most favorable overall trade-off” was removed and replaced with metric-specific interpretation.

#### 4.5.7. Statistical Analysis and Uncertainty Estimation

The quantitative results are reported as point estimates under a fixed event-level train/validation/test split. Event-level separation was preserved to avoid leakage between subsets because several station traces can correspond to the same earthquake. The final test subset contained 33 independent seismic events; therefore, small differences in F1 score, FAR, latency, or phase-picking error should be interpreted cautiously.

Because raw event-level prediction outputs for repeated independent runs were not available in the present revision, formal mean ± standard deviation values, bootstrap confidence intervals, and paired significance tests are not reported. Accordingly, the manuscript does not claim statistically proven superiority of the proposed framework over the strongest AI baseline. Instead, the reported values are interpreted as controlled benchmark point estimates and component-level performance trends under the defined Almaty-region waveform dataset and C1–C3 stress-test protocol.

For future fully reproducible statistical validation, event-level bootstrap confidence intervals should be computed for precision, recall, F1 score, and FAR, while paired M3–M4 comparisons should be performed using McNemar’s test for binary detection outcomes and the Wilcoxon signed-rank test for paired P- and S-pick absolute errors. These analyses require raw event-level prediction outputs and are therefore identified as a reproducibility requirement for subsequent validation rather than as completed statistical evidence in the present study.

The statistical reporting procedure used in the revised validation protocol is summarized in Table 14.

As shown in Table 14, the present study reports point estimates and explicitly avoids statistical-superiority claims. Repeated runs, bootstrap confidence intervals, and paired M3–M4 tests are identified as future uncertainty-analysis requirements rather than completed statistical evidence.

#### 4.5.8. Reproducibility Settings

All preprocessing, training, validation, quality-control, and evaluation settings were fixed before final test evaluation. The training subset was used only for model fitting. The validation subset was used for model selection, hyperparameter tuning, decision-threshold selection, and quality-control threshold selection. The test subset was used only for final reporting. The reproducibility-related settings are summarized in Table 15.

As shown in Table 15, the validation protocol separates the model fit, validation-based configuration, and final tests. This separation is necessary because the proposed structure includes both rules for neural inference and rules for decision-making after inference. Thus, the protocol allows for a more transparent interpretation of the presented results and reduces the risk of an optimistic bias.

The validation protocol described in this section serves as the basis for Section 5. The following section describes the comparison characteristics under standard and controlled noise conditions, behavior with reduced input and additional scattering, multitasking component analysis, and ablation results. All statements in the Results and their discussion should be limited to empirical waveform-oriented tests and the scenarios of controlled architectural stress testing described above.

## 5. Results

This section presents the experimental results obtained according to the validation protocol described in Section 4.5. The results are organized into four parts: comparative performance under standard and controlled noise conditions, architectural behavior under reduced-input and optional-branch scenarios, multi-task component analysis, and ablation-based assessment of the proposed framework. All results should be interpreted within the defined validation scope: the primary empirical benchmark is waveform-centered, while DAS, MEMS, and high-rate GNSS branches are evaluated only as controlled architectural scenarios rather than as a fully synchronized multimodal field deployment under real field conditions.

### 5.1. Comparative Performance of Existing Monitoring Approaches Under Standard and Noise-Perturbed Conditions

The first validation block compares four monitoring configurations under the standard waveform-centered benchmark and controlled degradation conditions. The evaluated methods are M1, a conventional trigger-based workflow; M2, a PhaseNet-like waveform-centered AI baseline; M3, an Earthquake Transformer-like joint detection-and-picking baseline; and M4, the proposed complete framework. The comparison is interpreted as an operational pipeline comparison rather than as a direct claim that the M4 waveform backbone is intrinsically superior to all existing neural architectures.

Under the standard-condition benchmark, the proposed framework achieved the highest reported F1 score and the lowest false-alarm rate among the evaluated configurations. M1 produced the weakest performance across the main metrics, whereas M2 and M3 substantially improved detection and phase-picking accuracy relative to the conventional workflow. The strongest AI baseline was M3, with an F1 score of 0.922, FAR of 0.069, detection latency of 196 ms, P-pick MAE of 38 ms, and S-pick MAE of 63 ms. M4 achieved a precision of 0.941, recall of 0.932, F1 score of 0.936, FAR of 0.051, detection latency of 173 ms, P-pick MAE of 31 ms, and S-pick MAE of 54 ms. These values indicate a moderate but consistent improvement across the reported metrics under the evaluated benchmark.

The standard-condition comparison is summarized in Table 16. The values are reported as point estimates under the fixed event-level split and are interpreted as controlled benchmark results rather than as formal statistical-superiority evidence.

The values in Table 16 are reported as point estimates under the fixed event-level split. They indicate that M4 produced the strongest reported benchmark values among the evaluated operational pipelines. However, because the final test subset contains only 33 independent seismic events and repeated-run outputs were not available, these differences are interpreted as controlled benchmark trends rather than statistically confirmed performance gains.

As shown in Table 16, M4 improved the reported point estimates for F1 score, FAR, latency, and phase-picking error relative to the strongest AI baseline. However, because the final test subset contains a limited number of independent events, the magnitude of these differences is interpreted conservatively and is not presented as statistically confirmed superiority.

**Figure 6:**
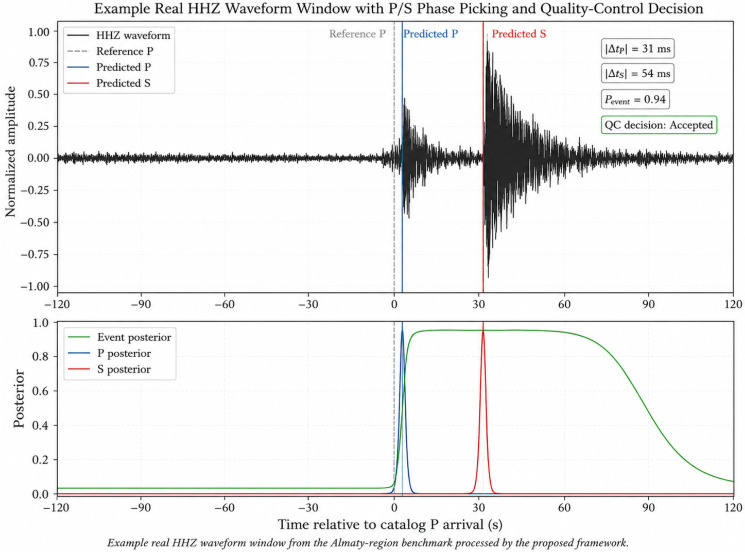
Example real HHZ waveform window from the Almaty-region benchmark processed by the proposed framework. The 240 s window is centered on the catalog-reported P-wave arrival and shows the waveform trace, reference P arrival, predicted P/S picks, posterior confidence, and final post-inference quality-control decision.

An example of a real HHZ waveform window processed by the proposed P/S phase-picking and quality-control workflow is shown in Figure 6.

Robustness was evaluated under the C1, C2, and C3 conditions using the same event-level test subset. C1 corresponds to the original preprocessed waveform windows, C2 to controlled medium-noise perturbation, and C3 to controlled low-SNR perturbation. All methods degraded as perturbation severity increased. The conventional workflow showed the largest decrease, from F1 = 0.816 in C1 to F1 = 0.653 in C3. M3 decreased from F1 = 0.922 to F1 = 0.804, whereas M4 decreased from F1 = 0.936 to F1 = 0.846. These values indicate greater point-estimate stability of the complete framework under the controlled perturbation protocol, but they are not interpreted as statistically confirmed superiority or as full validation under natural nonstationary seismic noise.

**Figure 7:**
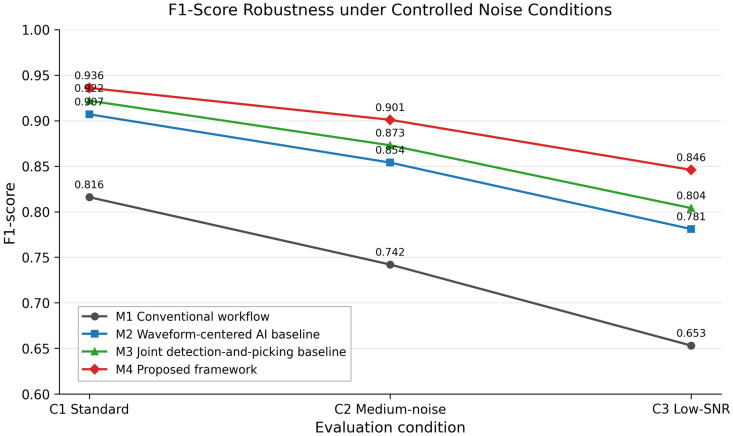
F1-score robustness of the compared seismic monitoring methods under C1 (standard), C2 (medium-noise) and C3 (low-SNR) conditions. The results show that all evaluated methods degraded as perturbation severity increased; however, the proposed framework preserved the highest F1 score across all three conditions and exhibited the smallest overall decline from C1 to C3.

The F1-score robustness comparison is summarized in Table 17 and illustrated in Figure 7. The figure shows the degradation trend from C1 to C3 while preserving the interpretation that controlled perturbation is a reproducible stress test rather than a complete representation of natural seismic-noise conditions.

As shown in Table 16 and Figure 7, the proposed framework produced the highest reported F1 score among all controlled evaluation conditions. The greatest practical difference was observed in the C3 condition, where a more significant contribution is expected from graph-aware refinement, post-inference quality control, and reduced-input logic. However, conditions C2 and C3 are reproducible stress tests and should not be considered complete models of unstable, location-dependent, or anthropogenic seismic noise.

### 5.2. Validation of the Unified Sensing and Processing Architecture

The second validation block checks whether the proposed architecture retains its functionality when working with complete, partial, and reduced-input data. The purpose of this analysis is not to demonstrate the full implementation of the real DAS–MEMS–HR-GNSS–seismic data fusion but to evaluate the architecture’s ability to support reliable waveform-centered monitoring behavior, even when additional auxiliary branches are unavailable, incomplete, or at the branch level of quality control.

**Figure 8:**
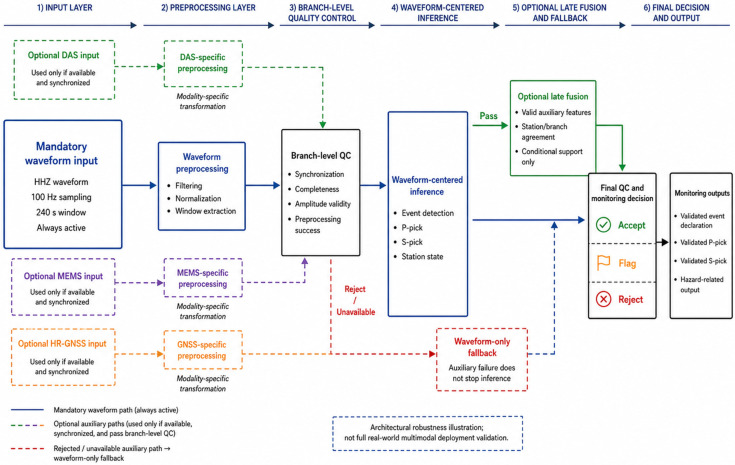
Reduced-input and optional-branch execution paths of the proposed waveform-centered monitoring framework, including waveform-only mode, optional auxiliary support, branch-level rejection, and fallback operation.

Figure 8 summarizes the reduced-input and optional-branch execution paths evaluated in this study. The workflow distinguishes waveform-only operation, optional auxiliary-branch support, branch-level rejection, late-stage fusion, final quality control, and waveform-only fallback when auxiliary inputs are unavailable or rejected.

The architecture-level results are summarized in Table 18. The reported results indicate that synchronized window construction was successful, with 98.7% of controlled branch-availability cases processed without manual correction, while 96.4% of auxiliary branches passed branch-level QC under the evaluated scenarios. The framework also preserved waveform execution after auxiliary-branch dropout and retained 94.8% of event decisions after single-branch removal. These results should be interpreted as evidence of reduced-input architectural robustness, not as empirical proof of a fully deployed multimodal field system.

The architecture-level percentages in Table 18 were obtained from controlled branch-availability, branch-rejection, and modality-dropout scenarios applied to the event-level validation and test protocol. These values quantify the execution behavior of the proposed framework under predefined auxiliary-branch availability rules and branch-level quality-control outcomes. They should not be interpreted as measurements from a fully synchronized real DAS–MEMS–HR-GNSS–seismic field deployment.

As shown in Table 18, the main architectural result is continuity of monitoring under reduced-input conditions. The optional auxiliary branches increased flexibility under the evaluated scenarios, but they were not required for waveform-centered inference. This distinction should be preserved throughout the Results and Discussion to avoid implying full empirical validation of simultaneous DAS, MEMS, high-rate GNSS, and seismic waveform fusion.

### 5.3. Performance of the AI-Assisted Multi-Task Detection, Phase-Picking and Hazard-Related Auxiliary Characterization Framework

The third validation block evaluates the contribution of the multi-task framework to event detection, P- and S-phase picking, graph-aware refinement, quality control, auxiliary support, and hazard-related output routing. The purpose of this subsection is to explain which components contributed to the reported performance and to avoid presenting the final result as the effect of a single unexplained predictor.

**Figure 9:**
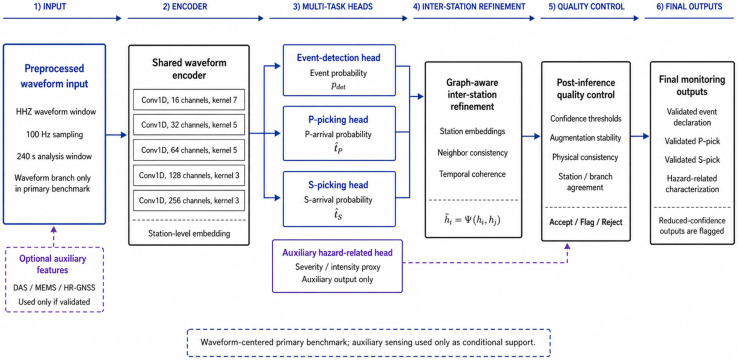
Simplified monitoring pipeline of the proposed multi-task waveform-centered monitoring framework, showing waveform inference, event detection, P/S phase picking, graph-aware refinement, post-inference quality control, and auxiliary hazard-related characterization.

Figure 9 presents the monitoring pipeline of the multi-task framework. It separates the waveform encoder, event-detection head, P-picking head, S-picking head, graph-aware refinement module, quality-control layer, and auxiliary hazard-related head.

As shown in Figure 9, the framework contains several analytically separable components. The waveform backbone produces the primary event and phase-related outputs, graph-aware refinement improves station-level consistency, the QC layer filters or flags unstable outputs, and the hazard-related head provides auxiliary rapid characterization after accepted or flagged monitoring outputs.

The component-level results are summarized in Table 19. The waveform backbone, alone, provided a competitive C1 F1 score of 0.921, indicating that the mandatory waveform branch is the primary source of predictive performance. Graph-aware refinement improved the C3 F1 score by 0.025 relative to the backbone-only configuration and reduced P- and S-pick MAE by 5 ms. The quality-control layer mainly reduced FAR, while optional auxiliary support provided the largest improvement under C3 when validated auxiliary input was available. These values support the interpretation that the final performance arises from complementary components rather than from a single isolated mechanism.

Hazard-related target: three-class catalog-magnitude severity proxy derived from event metadata; not a PGA, PGV, macroseismic-intensity, structural-damage, or full-hazard prediction.

Because repeated-run and event-level bootstrap outputs were not available in the present revision, the component-level differences in Table 19 are interpreted as point-estimate trends rather than statistically confirmed effects. This interpretation is consistent with the conservative statistical boundary defined in Section 4.5.7.

As shown in Table 19, graph-aware refinement and quality control contributed differently. Graph-aware refinement mainly improved temporal consistency and phase-picking behavior, whereas the QC layer mainly reduced the false-alarm rate and unstable outputs. The hazard-related head should be interpreted as an auxiliary rapid-characterization component; it does not replace full source inversion, rupture modeling, or comprehensive seismic hazard assessment.

### 5.4. Comparative Validation Under Reduced-Input and Ablation Conditions

The fourth validation block evaluates whether the claimed performance of the proposed architecture can be explained by specific components. The ablation analysis compares four configurations: only the basic structure for signal processing, the basic structure with graph-aware refinement, the basic structure with quality control, and the complete architecture. Such an analysis is necessary because M4 includes several operating levels beyond the output of the waveform.

**Figure 10:**
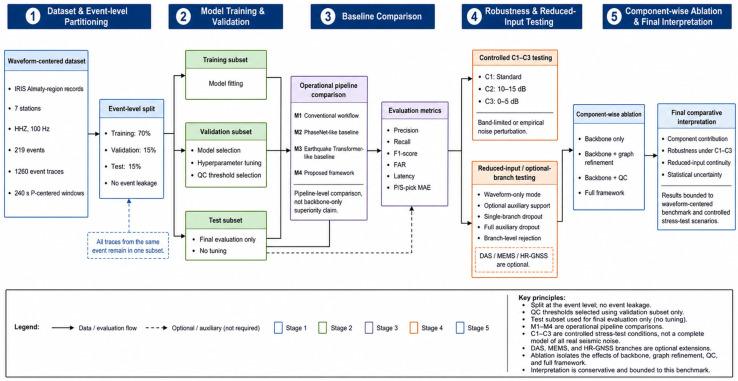
Comparative validation and ablation workflow, including event-level dataset partitioning, baseline comparison, controlled C1–C3 noise testing, reduced-input evaluation, optional-branch handling, and component-wise ablation.

Figure 10 summarizes the comparative validation and ablation workflow before the component-level ablation results are presented.

As shown in Figure 10, the ablation analysis uses the same event-level partitioning, metric definitions, and controlled noise conditions as the main benchmark. This consistency is important to ensure that differences among ablation configurations reflect component effects rather than changes in the data split or evaluation protocol.

The ablation results are summarized in Table 20. The backbone-only configuration achieved a C1 F1 score of 0.921 and C3 F1 score of 0.793. Adding graph-aware refinement increased the C1 F1 score to 0.928 and C3 F1 score to 0.818 while also reducing phase-picking errors. Adding QC without graph refinement produced a C1 F1 score of 0.929, C3 F1 score of 0.824, and FAR of 0.055. The full framework achieved a C1 F1 score of 0.936, C3 F1 score of 0.846, FAR of 0.051, latency of 173 ms, P-pick MAE of 31 ms, and S-pick MAE of 54 ms. These values are summarized in Table 20.

The ablation values in Table 20 are reported as point estimates under the fixed event-level validation protocol. They indicate that graph-aware refinement, quality control, and the complete framework contribute differently to the reported behavior. Graph-aware refinement mainly improves robustness and timing consistency, while post-inference quality control mainly reduces unstable detections and the false-alarm rate. The full framework combines these effects and provides the strongest reported point estimates. However, because repeated-run ablation experiments, bootstrap confidence intervals, and paired statistical tests were not available in the present revision, these values are interpreted as component-level trends rather than statistically confirmed differences between configurations.

### 5.5. Statistical Reporting Status and Interpretation Boundary

The numerical results reported in Table 16, Table 17, Table 18, Table 19 and Table 20 are point estimates under the fixed event-level validation protocol. Because the final test subset contains only 33 independent seismic events, these values are interpreted conservatively. No formal statistical-superiority claim is made. Repeated independent runs, event-level bootstrap confidence intervals, paired M3–M4 comparisons, and accepted/flagged/rejected QC-output statistics are identified as requirements for future reproducible validation.

Overall, the results indicate that the proposed framework improved the reported point estimates for detection quality, false-alarm control, latency, and phase-picking error under the waveform-centered Almaty benchmark and controlled C1–C3 stress-test conditions. The component and ablation analyses suggest that these improvements arise from complementary effects of waveform inference, graph-aware refinement, post-inference quality control, and reduced-input handling. However, the findings remain limited by the small independent test set, controlled auxiliary-branch scenarios, and the absence of synchronized real DAS–MEMS–HR-GNSS–seismic field deployment data.

## 6. Discussion

The results indicate that, with a deliberately more limited scope of validation, the waveform prediction accuracy is, indeed, one key element of reliable AI-assisted seismic monitoring, but so is the way the predictions are refined, filtered, and translated into operational decisions. The proposed framework achieved stronger point estimates than the evaluated conventional and AI-assisted reference pipelines for F1 score, false-alarm rate, latency, and P/S phase-picking error for the Almaty-region waveform benchmark and controlled C1–C3 stress-test protocol. However, the results are not interpreted as evidence of statistically confirmed superiority or as generalizations that are statistically significant.

The comparison among M1–M4 should be understood as an operational pipeline comparison. M1 represents a conventional trigger-based workflow; M2 represents a waveform-based AI reference workflow inspired by PhaseNet-like processing; M3 represents an Earthquake Transformer-like joint detection-and-picking workflow; and M4 represents the complete proposed pipeline with waveform inference, graph-aware refinement, post-inference quality control, reduced-input fallback, and optional auxiliary-branch handling. Therefore, the results do not show that the M4 waveform encoder is inherently superior to all existing neural pickers. Rather, they suggest that combining waveform inference with explicit refinement and decision-level reliability control can improve point-estimate monitoring behavior under the evaluated protocol [3,4].

Based on the ablation results, the observed behavior cannot be explained by one component. The primary predictive basis is the waveform backbone. Under the strongest controlled degradation condition, which is similar to recent studies with multi-station and graph-aware approaches that found station-level context can help with phase picking, association and monitoring consistency [7,10,12], graph-aware refinement enhances station-level consistency. The quality-control layer serves a different purpose: its primary intent is not to boost the raw predictive power but to filter or flag unreliable detections and physically inconsistent picks for final monitoring output. This distinction is key, as raw neural posterior probabilities might be sensitive to a low SNR, domain shifts, incomplete station coverage, or heterogeneous sensing conditions [8,16].

The auxiliary branches are optional and should be treated with care. The present study does not provide empirical validation of simultaneous real DAS–MEMS–HR-GNSS–seismic fusion. The primary empirical benchmark is taken from the real waveform-centered HHZ records of the Almaty seismic region. DAS, MEMS, and high-rate GNSS branches are optional extensions of the architecture and are tested only in limited branch-availability, reduced-input, fallback, and stress-test scenarios. The results do not show a fully deployed real-world multimodal monitoring system, but this does not contradict the feasibility of a waveform-centered framework with optional auxiliary support.

The conservative design of the fusion is justified by the physical difference between the modalities of the sensors. The conventional seismic waveforms, DAS strain or strain-rate measurements, MEMS acceleration measurements, and high-rate GNSS displacement-related records are not in the same units. They have different sampling rates, transfer functions, amplitude units, spatial supports, timing precisions, dynamic ranges, installation sensitivities, and noise behaviors. For this reason, the framework does not combine all the modalities into a single raw waveform tensor. Rather, it applies modality-specific preprocessing and permits auxiliary branches to contribute after quality control at the branch level. This approach is similar to the recent DAS, MEMS, and high-rate GNSS studies that demonstrate the need for specific representation and handling of reliability [5,6,13,14,15].

The C1–C3 perturbation protocol is a repeatable test to compare the robustness of the system but does not fully capture natural seismic noise. Seismic noise in nature may occur in the form of colored, nonstationary, site-dependent, source-dependent, anthropogenic, and environmental processes. Hence, the observed stability under C2 and C3 should be viewed as ‘controlled stress-test behavior’. More validation in naturally noisy low-SNR records, in other tectonic locations, and over other station networks is needed before claims of robustness or transferability can be made.

The auxiliary hazard-related head is interpreted conservatively as well. In the current study, it is applied as a proxy for the severity of events, in the form of catalog magnitude, following event declaration and phase picking. It is not a prediction of PGA, PGV, macroseismic intensity, structural damage, rupture properties, or comprehensive seismic hazard assessment. Hence, this output should be used as an auxiliary monitoring indicator only and not as a substitute for source inversion, ground-motion simulation, or a complete seismic hazard assessment.

The limited number of independent test events remains an important constraint. The event-level 70/15/15 split does not result in leakage of the information in the test set at the trace level, but the test set, itself, contains only 33 independent earthquakes. This reduces the statistical power of the comparison and makes it more likely that variations in F1 score or the false-alarm rate are due to a few event-level decisions. For this reason, the revised manuscript reports only point estimates and does not claim statistically proven superiority.

This study should be interpreted as a conservative framework-level evaluation rather than as evidence of a universally superior seismic predictor. The results within the evaluated Almaty-region waveform benchmark indicate that a compact waveform backbone, coupled with graph-aware refinement, quality control based on validation, and a reduced-input fallback, can enhance operational point estimates. Larger cross-domain waveform datasets, natural low-SNR records, synchronized real DAS/MEMS/HR-GNSS/seismic deployments, and complete uncertainty quantification are the next areas of research.

## 7. Conclusions

This study developed and evaluated an AI-assisted, waveform-centered framework for seismic monitoring with post-inference quality control. The framework incorporates waveform-based event detection and P/S phase picking, graph-aware inter-station refinement, reduced-input fallback, and auxiliary magnitude-related rapid characterization. The primary empirical validation is based on real waveform-centered IRIS/EarthScope records from the Almaty seismic region, while optional DAS, MEMS, and high-rate GNSS branches are included as architectural extensions but were not validated as a fully synchronized real-world multimodal field deployment.

Under the standard-condition benchmark, the proposed complete framework produced the strongest reported point estimates among the evaluated operational pipelines: precision = 0.941, recall = 0.932, F1 score = 0.936, false alarm rate = 0.051, detection latency = 173 ms, P-pick MAE = 31 ms, and S-pick MAE = 54 ms. Under the controlled low-SNR stress-test condition, it retained an F1 score of 0.846. These results indicate improved point-estimate monitoring behavior under the defined benchmark and controlled perturbation protocol, but they are not presented as statistically confirmed superiority or universal generalization.

The results of the ablation analysis indicate that the final behavior is not determined by a single predictor but by complementary components. Graph-aware refinement primarily increases the consistency of the station-level and phase-picking behavior, and the post-inference quality-control layer primarily decreases unstable detections and the false-alarm rate. Its greatest point-estimate benefit is given in the worst controlled condition, and when no auxiliary branches exist or are accepted, waveform-only fallback allows for operational continuity.

This study has clear limitations. The empirical benchmark is based on the real HHZ waveform records of seven stations in the Almaty region but does not include a synchronized real DAS–MEMS–HR-GNSS–seismic field dataset. The C1–C3 perturbation protocol is reproducible stress testing but does not fully represent natural colored and nonstationary seismic noise. The final test set consists of only 33 independent events, and therefore, all numerical values are understood as point estimates and not as statistically confirmed superiority claims.

Future work should focus on four directions: (1) validation with larger cross-domain waveform datasets; (2) testing under naturally noisy, low-SNR in-field conditions; (3) synchronized real DAS/MEMS/HR-GNSS/ seismic field deployment; and (4) rigorous uncertainty estimation using multiple runs of independent datasets, event-level bootstrap confidence intervals, paired M3–M4 tests, and accepted/flagged/rejected QC-output statistics. These steps are required before the framework can be presented as a deployable, real-world multimodal seismic monitoring system.

## Figures and Tables

**Table 1:** Functional decomposition of the proposed multi-sensor seismic monitoring framework.

Subsystem	Function
Primary waveform acquisition layer	Acquisition and preprocessing of seismic waveform windows for event detection and P/S phase picking
Auxiliary sensing layer	Conditional ingestion of DAS, MEMS, and high-rate GNSS observations when synchronized and quality-controlled measurements are available
Preprocessing and synchronization block	Demeaning, detrending, filtering, normalization, resampling (where applicable), window extraction, and branch-level alignment
Distributed forwarding layer	Local screening and confidence-aware forwarding of candidate waveform or auxiliary segments
Waveform inference backbone	Event detection and P/S phase probability estimation from the mandatory seismic waveform branch
Graph-aware refinement block	Inter-station consistency refinement using station-level embeddings or prediction states
Fusion and decision block	Conditional integration of valid branch outputs and generation of monitoring decisions
Quality-control block	Confidence estimation, augmentation-stability checking, physical consistency filtering, and uncertain-output flagging
Reduced-input fallback logic	Continuation of waveform-only inference when auxiliary branches are unavailable or rejected
Hazard-related auxiliary characterization head	Auxiliary rapid characterization output after quality-controlled event declaration and phase picking

**Table 2:** Principal parameters of the signal representation and preprocessing stage.

Parameter	Description	Value or Status in the Study
L	Analysis-window length	240 s
$f_{s}$	Sampling frequency of waveform input	100 Hz
Waveform channel	Empirical waveform input channel	HHZ
$C_{w}$	Number of waveform input channels	1 in the reported HHZ benchmark; 3 only as an optional extension
$M_{aux}$	Optional auxiliary modalities	DAS, MEMS, and HR-GNSS when available
$\Phi_{w}(\cdot )$	Waveform preprocessing and encoding operator	Demeaning, detrending, filtering, normalization, windowing, and temporal encoding
$\Phi_{DAS}(\cdot )$	DAS transformation operator	Spatial–temporal panel representation; optional branch
$\Phi_{MEMS}(\cdot )$	MEMS transformation operator	Acceleration-related representation; optional branch
$\Phi_{GNSS}(\cdot )$	HR-GNSS transformation operator	Displacement- or velocity-related representation; optional branch
$B=[f_{low},f_{high}]$	Effective band-pass range	1.0–45.0 Hz zero-phase fourth-order Butterworth band-pass filter
N(⋅)	Amplitude-normalization operator	Applied before waveform inference
F(⋅)	Feature- or decision-construction operator	Waveform-only in primary benchmark; conditional fusion in optional-branch scenarios
Branch QC	Auxiliary-branch validity screening	Synchronization, completeness, amplitude validity, and preprocessing success

**Table 3:** Architecture of the waveform inference backbone.

Block	Operation	Channels	Kernel Size	Output Role
Input	Preprocessed waveform window	1 in the reported HHZ benchmark; 3 when three-component records are available	-	Seismic waveform input
Encoder block 1	Conv1D + BatchNorm + ReLU	16	7	Low-level waveform features
Encoder block 2	Conv1D + BatchNorm + ReLU	32	5	Local arrival patterns
Encoder block 3	Conv1D + BatchNorm + ReLU	64	5	Mid-level temporal features
Encoder block 4	Conv1D + BatchNorm + ReLU	128	3	High-level event features
Encoder block 5	Conv1D + BatchNorm + ReLU	256	3	Station-level embedding
Decoder	Upsampling + Conv1D	16–128	3	Sample-wise probability reconstruction
Output features	Shared temporal representation	-	-	Input to task-specific heads

**Table 4:** Loss weights used in the multi-task training objective.

Loss Term	Weight	Rationale
Detection loss	λ_det_ = 1.0	Event declaration accuracy
Phase-picking loss	λ_pick_ = 2.0	Higher importance of P/S arrival-time precision
Hazard-related loss	λ_haz_ = 0.5	Auxiliary rapid-characterization output

**Table 5:** Training hyperparameters used for the learning-based models.

Hyperparameter	Value
Optimizer	Adam
Initial learning rate	10^−3^
Batch size	32
Maximum epochs	100
Early stopping patience	10 epochs
Weight decay	10^−5^
Learning-rate scheduler	ReduceLROnPlateau
Model selection	Best validation F1 score and validation loss
Run reporting	Point-estimate reporting under a fixed event-level split
Threshold tuning	Validation subset only

**Table 6:** Quality-control thresholds used in the post-inference decision layer.

QC Criterion	Symbol	Value	Selection Procedure
Event confidence threshold	τ_q,det_	0.6	Selected based on validation F1 score
P-pick confidence threshold	τ_q,P_	0.55	Selected using validation P-pick MAE and valid pick rate
S-pick confidence threshold	τ_q,S_	0.50	Selected using validation S-pick MAE and valid pick rate
P-pick stability threshold	τ_σ,P_	0.03 s	Based on validation pick-dispersion distribution
S-pick stability threshold	τ_σ,S_	0.05 s	Based on validation pick-dispersion distribution
Minimum S–P interval	Δ_min_	0.1 s	Regional acquisition and phase-ordering constraint
Maximum S–P interval	Δ_max_	30 s	Regional station-geometry constraint
Minimum agreement score	Q_agg_	0.50	Validation-based consistency threshold

**Table 7:** Summary of the waveform-centered empirical dataset used in the primary validation.

Parameter	Value
Region	Almaty seismic region and surrounding Central Asian area
Reference coordinates	43.22° N, 76.92° E
Coverage radius	3.0° approximately 333 km
Data source	FDSN-compatible IRIS/EarthScope waveform and metadata services
Observation period	1 January 2023–31 December 2024
Station networks	QZ, KR, and KC
Stations	TARG, ARLS, BAET, JNKS, BTLS, SHLS, and TLG
Channel	HHZ
Sampling rate	100 Hz
Number of seismic events	219
Number of event traces	1260
Window length	240 s
Window position	120 s before and 120 s after the reported P-wave arrival
Primary empirical validation	Real waveform-centered benchmark
Auxiliary DAS/MEMS/HR-GNSS branches	Optional architectural extensions evaluated through controlled reduced-input and branch-availability scenarios

**Table 8:** Station-network affiliation and available station coordinates used for documentation of the regional waveform corpus.

Station	Network Code	Network Description	Latitude (° N)	Longitude (° E)	Operational Note
TLG	QZ	Kazakhstan SEME seismic network	43.249	77.224	Waveform benchmark station
BTLS	QZ	Kazakhstan SEME seismic network	45.041	74.046	Waveform benchmark station
SHLS	QZ	Kazakhstan SEME seismic network	43.161	79.885	Waveform benchmark station
ARLS	KR	Kyrgyz Digital Network	41.853	74.335	Waveform benchmark station
BAET	KR	Kyrgyz Digital Network	41.0899	75.0148	FDSN metadata: elevation 3015 m; start date 9 July 2024; shorter 2023–2024 coverage
JNKS	KR	Kyrgyz Digital Network	41.053	75.626	Waveform benchmark station
TARG	KC	Central Asian Seismic Network of CAIAG	41.729	77.805	Waveform benchmark station

**Table 9:** Event-level partitioning of the waveform-centered corpus.

Subset	Events	Event Traces	Purpose
Training	153	approximately 882	Model fitting
Validation	33	approximately 189	Hyperparameter selection, model selection, and QC threshold tuning
Test	33	approximately 189	Final evaluation only
Total	219	1260	Complete waveform-centered corpus

**Table 10:** Monitoring configurations compared in the validation protocol.

Method	Configuration	Main Role in the Study	Main Components
M1	Conventional trigger-based workflow	Classical operational reference	Trigger-based event declaration and classical phase-picking logic
M2	Waveform-centered AI baseline	Deep learning waveform reference	PhaseNet-like waveform inference for phase-related probability estimation
M3	Joint detection-and-picking AI baseline	Strong AI reference model	Earthquake Transformer-like joint event detection and phase-picking workflow
M4	Proposed quality-controlled framework	Complete operational framework	Waveform inference, graph-aware refinement, quality control, reduced-input fallback, and optional auxiliary-branch support

**Table 11:** Quantitative definition of the controlled evaluation conditions.

Condition	Description	Target SNR Range	Purpose
C1	Original preprocessed test windows without additional perturbation	Original SNR	Standard-condition benchmark
C2	Test windows with controlled band-limited noise perturbation	10–15 dB	Medium-noise robustness evaluation
C3	Test windows with stronger controlled band-limited noise perturbation	0–5 dB	Low-SNR robustness evaluation

**Table 12:** Reduced-input and optional-branch evaluation scenarios.

Scenario	Available Input Configuration	Branch-Handling Rule	Purpose
Waveform-only mode	Primary seismic waveform branch only	Auxiliary branches are not used	Baseline valid execution mode of the proposed framework
Optional-branch mode	Waveform branch plus available auxiliary branches	Auxiliary information is used only after branch-level quality control	Evaluation of conditional auxiliary support
Single-branch dropout	Waveform branch plus remaining valid auxiliary branches	One auxiliary branch is removed or rejected	Testing tolerance to partial auxiliary input loss
Full auxiliary dropout	Waveform branch only after all auxiliary branches are unavailable or rejected	System returns to waveform-only fallback mode	Testing reduced-input operational continuity
Branch-level rejection	Waveform branch plus auxiliary branches with failed QC	Failed auxiliary branches are excluded from fusion	Preventing unreliable auxiliary information from affecting final decisions

**Table 13:** Evaluation metrics used in the validation protocol.

Metric	Definition	Interpretation
Precision	$\frac{TP}{\left(TP+FP\right)}$	Fraction of declared events that were correct
Recall	$\frac{TP}{\left(TP+FN\right)}$	Fraction of reference events that were detected
F1 score	$\frac{2PR}{\left(P+R\right)}$	Harmonic mean of precision and recall
FAR	$\frac{FP}{N_{noise}}$	False event declarations per evaluated noise-only window
Detection latency	${\hat{t}}_{det}-t_{det}^{ref}$	Delay between the reference event time and the model-based event declaration
P-pick MAE	Mean absolute difference between predicted and reference P-arrival times	Average P-arrival timing error
S-pick MAE	Mean absolute difference between predicted and reference S-arrival times	Average S-arrival timing error
Hazard MAE	Mean absolute difference between predicted and reference hazard-related values	Average error of the auxiliary hazard-related regression output
Hazard macro-F1	Mean F1 score across all hazard classes	Classification accuracy for categorical hazard-related output

**Table 14:** Statistical interpretation and future uncertainty-analysis requirements.

Analysis Component	Applied Procedure	Purpose
Event-level data separation	Applied	Prevent leakage between training, validation, and test subsets
Current metric reporting	Point estimates under a fixed event-level split	Avoid unsupported statistical-superiority claims
Current statistical claim	No formal statistical-superiority claim	Maintain conservative interpretation of limited test data
Future repeated runs	Required for subsequent validation	Quantify run-to-run variability
Future bootstrap confidence intervals	Required for subsequent validation	Estimate uncertainty for precision, recall, F1 score, FAR, and phase-picking errors
Future paired M3–M4 comparison	Required for subsequent validation	Assess whether observed gains are statistically meaningful
Interpretation rule	Results discussed as benchmark trends	Avoid overclaiming from 33 independent test events

**Table 15:** Reproducibility-related settings of the revised validation protocol.

Component	Fixed Setting or Reporting Rule	Purpose
Data partitioning	Event-level 70/15/15 split	Prevent trace-level leakage between subsets
Training subset	Used only for model fitting	Avoid contamination of validation and test results
Validation subset	Used for model selection, hyperparameter tuning, and QC threshold selection	Ensure that tuning is separated from final testing
Test subset	Used only for final reporting	Prevent optimistic performance estimates
Preprocessing	Same preprocessing pipeline for all learning-based configurations	Ensure fair comparison
Noise conditions	C1, C2, and C3 fixed before final evaluation	Ensure reproducible robustness testing
QC thresholds	Selected based on validation data and fixed before testing	Avoid test-set threshold tuning
FAR definition	$\frac{N_{FP}}{N_{noise}}$	Use one consistent false-alarm standard
Statistical reporting	Point estimates under a fixed event-level split	Avoid unsupported statistical-superiority claims
Comparison type	Operational pipeline comparison	Clarify that M4 is evaluated as a complete monitoring pipeline

**Table 16:** Standard-condition comparison of M1–M4.

Method	Precision	Recall	F1 Score	FAR	Detection Latency (ms)	P-Pick MAE (ms)	S-Pick MAE (ms)
M1—Conventional workflow	0.841	0.792	0.816	0.146	428	96	148
M2—Waveform-centered AI baseline	0.914	0.901	0.907	0.081	214	42	69
M3—Joint detection-and-picking baseline	0.926	0.918	0.922	0.069	196	38	63
M4—Proposed framework	0.941	0.932	0.936	0.051	173	31	54

**Table 17:** Noise robustness comparison of M1–M4 using F1 score.

Method	C1—Standard	C2—Medium Noise	C3—Low SNR
M1—Conventional workflow	0.816	0.742	0.653
M2—Waveform-centered AI baseline	0.907	0.854	0.781
M3—Joint detection-and-picking baseline	0.922	0.873	0.804
M4—Proposed framework	0.936	0.901	0.846

**Table 18:** Controlled branch-availability and reduced-input execution results of the proposed architecture.

Architectural Function	Observed Validation Result	Operational Implication
Synchronized window construction	98.7% of admissible multimodal windows were aligned without manual correction	Stable common analysis input for heterogeneous sensing
Branch-specific preprocessing	100% waveform execution; 96.4% auxiliary-branch acceptance after branch-level QC	Auxiliary data used without forcing a misleading common raw format
Reduced-input execution	100% successful fallback after auxiliary-branch dropout; 94.8% of event decisions preserved after single-branch removal	No pipeline collapse under missing modalities
Confidence-aware forwarding	27.4% of low-quality candidate windows were filtered before final decision output	Lower downstream instability and reduced false triggering
Late fusion and decision block	Under C3, F1 score increased from 0.824 in backbone + QC mode to 0.846 in the full framework when validated auxiliary input was available	Conditional auxiliary support provided the largest point-estimate gain under C3 in the controlled branch-availability scenario
Hazard-head activation routing	91.6% of QC-accepted detections were forwarded to hazard-related output; 8.4% were flagged as uncertain	Downstream uncertainty handled explicitly

**Table 19:** Analytical components and measured contribution of the proposed multi-task framework.

Component	Direct Results	Quantified Effect
Event-detection head	Stable waveform-centered base inference	Backbone-only F1 score = 0.921 under C1
P-phase picking head	Highest timing stability among phase tasks	P-pick MAE = 31 ms in full framework
S-phase picking head	Higher noise sensitivity than P-phase task	S-pick MAE = 54 ms in full framework
Graph-aware refinement	Improved temporal consistency across stations	Relative to backbone only: C3 F1 score + 0.025; P-pick MAE—5 ms; S-pick MAE—5 ms
Quality-control layer	Removed unstable detections and inconsistent picks	FAR reduced from 0.074 to 0.055 relative to backbone only
Auxiliary sensing support	Improved hardest-condition robustness when validated inputs were available	C3 F1 score increased from 0.824 in backbone + QC mode to 0.846 in full framework
Hazard-related head	Preserved downstream rapid classification usability	Macro-F1 = 0.84 in full-input mode; 0.80 in reduced-input mode

**Table 20:** Ablation study of the proposed framework.

Configuration	C1 F1 Score	C3 F1 Score	FAR	Detection Latency (ms)	P-Pick MAE (ms)	S-Pick MAE (ms)
Backbone only	0.921	0.793	0.074	189	39	64
Backbone + graph refinement	0.928	0.818	0.067	183	34	59
Backbone + QC	0.929	0.824	0.055	186	37	60
Full framework	0.936	0.846	0.051	173	31	54

## Data Availability

The primary waveform data used in this study were obtained from open-access IRIS/EarthScope Data Services through FDSN-compatible services. Event metadata and waveform records can be retrieved from the corresponding public data services using the station list, time period, channel, and regional selection criteria reported in this manuscript. Derived train/validation/test splits, preprocessing scripts, model configuration files, and processed prediction outputs are available from the corresponding author upon reasonable request. Patent-sensitive diagrams, unpublished implementation details, and auxiliary architectural materials are not publicly released at this stage.

## Abbreviations

The following abbreviations are used in this manuscript:

AI	Artificial Intelligence
DAS	Distributed Acoustic Sensing
FAR	False-Alarm Rate
F1-score	Harmonic Mean of Precision and Recall
GNSS	Global Navigation Satellite System
HR-GNSS	High-Rate Global Navigation Satellite System
IRIS	Incorporated Research Institutions for Seismology
MAE	Mean Absolute Error
MEMS	Micro-Electro-Mechanical System
QC	Quality Control
SNR	Signal-to-Noise Ratio
